# CardioCLIP: sequential multimodal prediction of echocardiographic abnormalities and natriuretic peptide elevation from electrocardiogram and chest radiographs

**DOI:** 10.1093/ehjdh/ztag158

**Published:** 2026-10-01

**Authors:** Satoki Shibata, Makoto Nishimori, Yu Nishihara, Eiichi Maeda, Masaaki Iiyama, Masakazu Shinohara

**Affiliations:** Kobe University School of Medicine, Kobe, Japan; Division of Molecular Epidemiology, Kobe University Graduate School of Medicine, 7-5-2, Kusunoki-cho, Chuo-ku, Kobe 650-0017, Japan; Division of Cardiovascular Medicine, Department of Internal Medicine, Kobe University Hospital, Kobe, Japan; Division of Medical Informatics, Department of Internal Related, Kobe University Graduate School of Medicine, Kobe, Japan; Faculty of Data Science, Shiga University, Shiga, Japan; Division of Molecular Epidemiology, Kobe University Graduate School of Medicine, 7-5-2, Kusunoki-cho, Chuo-ku, Kobe 650-0017, Japan

**Keywords:** Sequential multimodal learning, Electrocardiography, Chest X-ray, Echocardiographic abnormalities, Natriuretic peptides, Deep learning

## Abstract

**Aims:**

Diagnostic reasoning in clinical practice is inherently sequential and multimodal, with physicians integrating heterogeneous examinations under constraints such as missing data and variable test order. However, most multimodal machine learning models assume simultaneous availability of all modalities and fail to reflect this workflow.

**Methods and results:**

We propose CardioCLIP, a sequential multimodal framework that embeds electrocardiograms (ECGs) and chest X-rays (CXRs) into a shared pathological representation space. The model consists of (i) a self-supervised embedding stage aligning ECG–CXR pairs from the same patient and day, and (2) a gated recurrent unit-based prediction stage that estimates clinical indicators as data are sequentially observed. The model was trained on 9458 patients from a single tertiary centre and evaluated for B-type natriuretic peptide (BNP), left ventricular ejection fraction (LVEF), and *E*/*e*′. External validation was conducted using MIMIC-IV.

CardioCLIP achieved strong cross-modal alignment and improved discrimination for selected echocardiographic abnormalities and natriuretic peptide elevation (AUC: 0.90 for BNP > 100 pg/mL, 0.87 for LVEF < 50%, and 0.80 for *E*/*e*′ > 15). It also showed discrimination for subsequent severe BNP elevation (BNP > 400 pg/mL within 3 years) with an AUC of 0.79. External validation in MIMIC-IV showed an AUC of 0.89 for N-terminal pro-BNP elevation, supporting potential transportability for this endpoint.

**Conclusion:**

CardioCLIP demonstrated the feasibility of order-flexible sequential multimodal prediction. By leveraging routinely acquired ECG and CXR data, it may provide a non-invasive adjunct for identifying echocardiographic abnormalities and natriuretic peptide elevation in cardiovascular care, subject to prospective clinical validation.

## Graphical Abstract

**Graphical Abstract:**
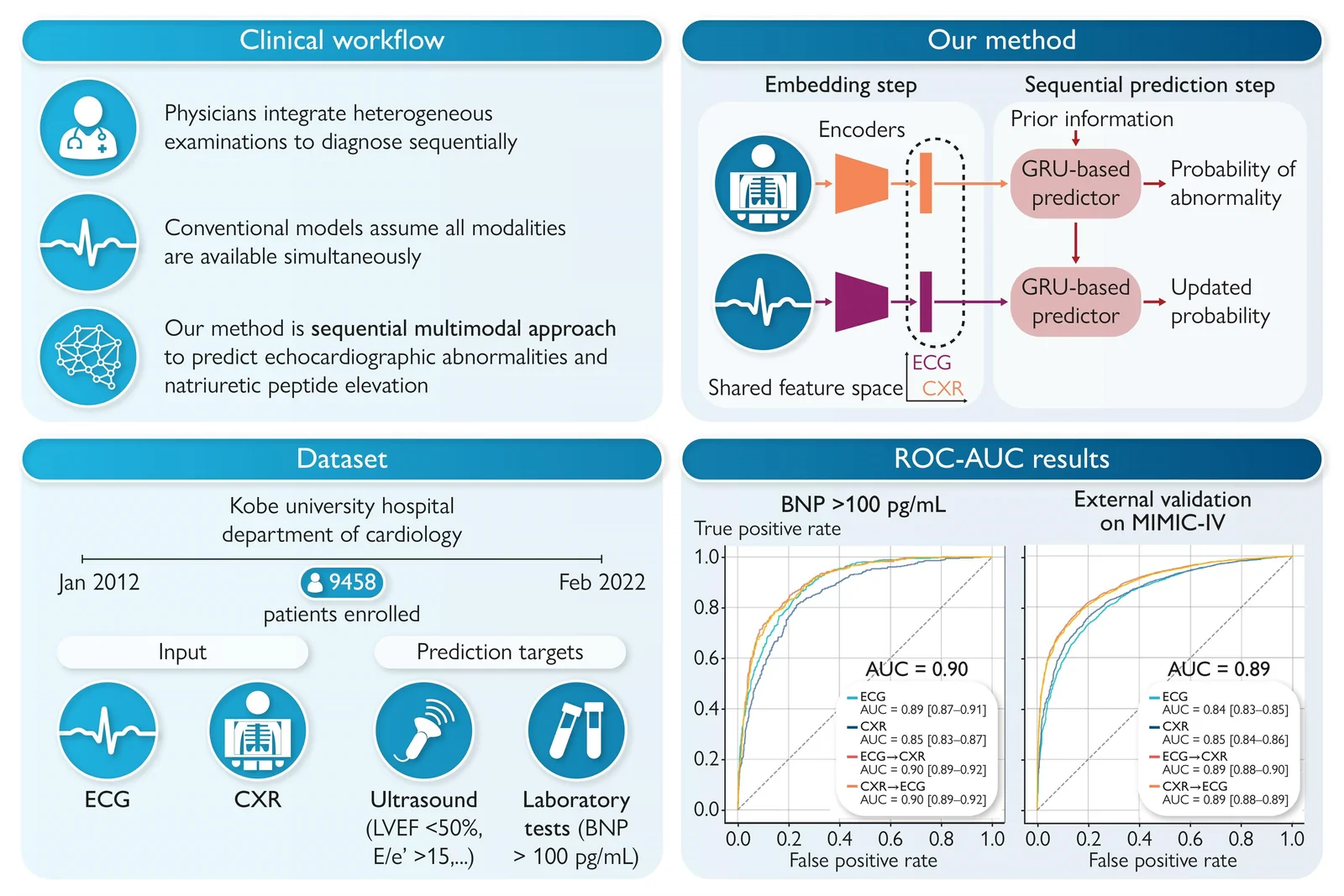

## Introduction

In recent years, the application of machine learning in healthcare has advanced rapidly, with numerous studies demonstrating its effectiveness in diagnosis and prognosis prediction.^1,2^ Prior work has proposed predictive models based on single modalities—such as medical imaging, electrocardiograms (ECGs), and laboratory data^3–6^—as well as multimodal models integrating multiple data sources.^7–11^ However, many existing approaches do not adequately reflect the diagnostic reasoning process of clinicians in real-world practice.

In clinical settings, physicians do not rely on a single test result but instead evaluate multiple examinations sequentially, integrating heterogeneous information to infer underlying pathophysiology. This process is inherently sequential and multimodal. Nevertheless, current multimodal models have several limitations. First, most models require all modalities to be available simultaneously, which is inconsistent with the stepwise nature of clinical workflows. Although methods that tolerate missing modalities have been proposed,^12,13^ these approaches are largely limited to homogeneous imaging modalities, and sequential missing-modality frameworks for heterogeneous data—such as ECGs and chest X-rays (CXRs)—remain underexplored. Second, there is an unresolved design challenge regarding the optimal stage at which multimodal information should be integrated.^14–16^ Third, many models require retraining when new modalities are added, limiting scalability and practical applicability.

To address these challenges, contrastive learning^17^ has emerged as an effective approach for extracting shared representations across modalities. In particular, Contrastive Language–Image Pre-training (CLIP)^18^ has gained attention by mapping text and images into a shared semantic space, and its applications in the medical domain have expanded to image–text^19,20^ and ECG–text pairs.^21,22^ Recent work has also demonstrated that contrastive learning can be applied to ECG–CXR pairs to capture shared pathological features.^23^

In the cardiovascular domain, screening based on echocardiographic parameters and natriuretic peptide levels such as left ventricular ejection fraction (LVEF), *E*/*e*′ ratio, and B-type natriuretic peptide (BNP) is essential. If these indicators could be estimated sequentially from routinely acquired ECGs and CXRs, it would enable non-invasive and cost-effective clinical assessment.^24–27^

In this study, we propose CardioCLIP, a framework that embeds heterogeneous modalities—ECGs and CXRs—into a shared pathological representation space using CLIP.^18^ As illustrated in *Figure 1*, CardioCLIP consists of two stages: (i) an embedding stage that maps ECG and CXR data into a unified representation space, and (ii) a sequential prediction stage in which the learned embeddings are used for downstream clinical prediction via a gated recurrent unit (GRU).^28,29^ This design enables flexible prediction of underlying pathology with either unimodal input or different modality-input order. Through this approach, we aim to provide an order-flexible framework for prediction in cardiovascular care.

**Figure 1:**
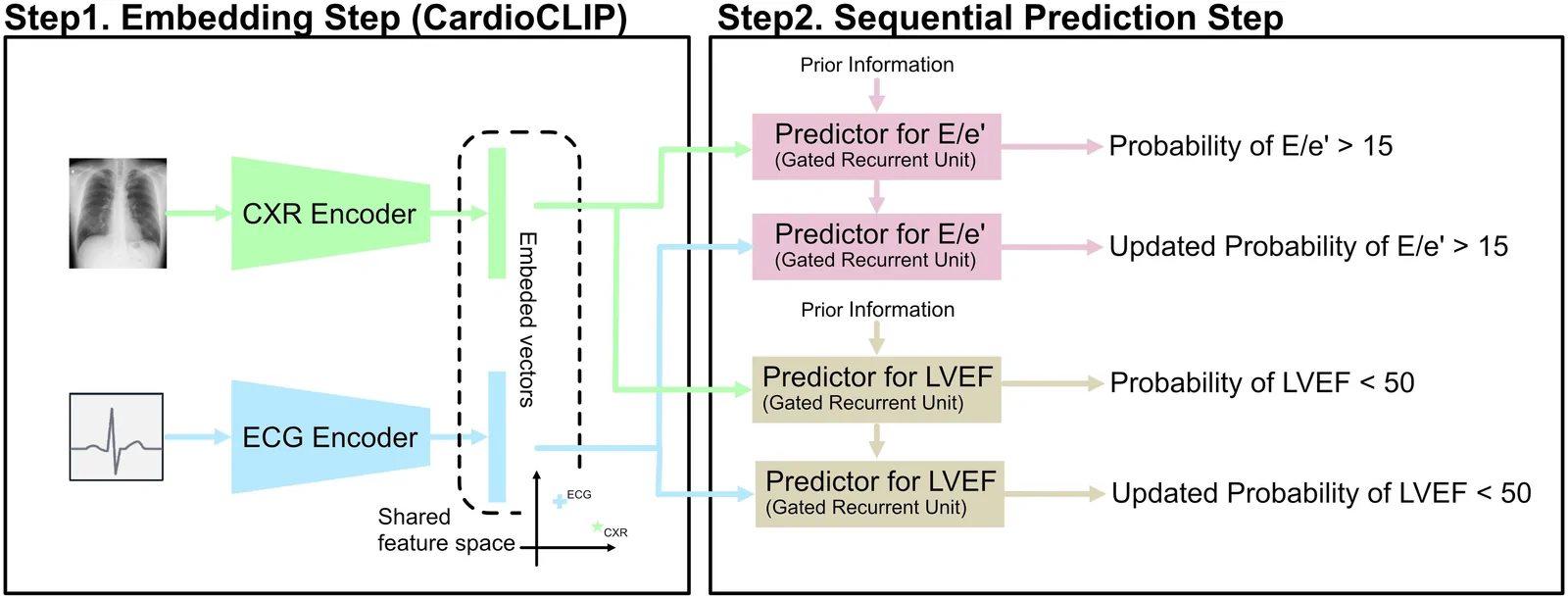
Sequential prediction with CardioCLIP. In Step 1, electrocardiogram and chest X-ray data are each encoded by their respective encoders and embedded into a shared feature space representing the underlying disease state. In Step 2, the embedded vectors are sequentially input into a predictor, which detects abnormalities in clinical indices such as left ventricular ejection fraction and the *E*/*e*′ ratio.

## Methods

### Study design, data sources, and study population

This study included 9458 patients who visited the Department of Cardiology at Kobe University Hospital between January 2012 and February 2022, and who had both an ECG and a frontal CXR acquired on the same day, yielding a total of 24 135 paired samples.

As illustrated in *Figure 1*, the proposed framework consists of a two-stage analytical pipeline. In Step 1, pre-training was performed using a large set of unlabelled ECG–CXR pairs. In Step 2, downstream training and evaluation were conducted using labelled data to predict clinical indicators, such as echocardiographic parameters and laboratory measurements.

In Step 1, the data from all 9458 patients described above were split and used for training, validation, and testing. For Step 2, patients were selected based on the availability of corresponding outcome data for each prediction task (echocardiographic and laboratory parameters). For echocardiographic data, the examination performed closest to the date of ECG and CXR acquisition was used, resulting in 8262 eligible patients. Similarly, for laboratory data, measurements obtained closest to the imaging date were selected, and all 9458 patients were included. The detailed characteristics of the dataset are presented in *Table 1*.

**Table 1:** Baseline demographics and clinical characteristics of the study population

Patients, *n*	9458
ECG–X-ray pairs, *n*	24 135
Age, years	69.6 ± 22.1
Age groups, *n* (%)	
0–17 years	57 (0.6%)
18–60 years	2530 (26.7%)
61–70 years	2329 (24.6%)
71–80 years	2881 (30.4%)
81 + years	1661 (17.5%)
Gender, *n* (%)	
Male	5717 (60.4%)
Female	3741 (39.5%)
Height, cm	159.6 ± 17.2
Body weight, kg	58.0 ± 17.9
Body mass index, kg/m^2^	22.6 ± 5.1
Haemoglobin, g/dL	12.0 ± 3.20
HbA1c, %	6.10 ± 1.10
Blood urea nitrogen (BUN), mg/dL	18.3 ± 12.1
eGFR (creatinine-based), mL/min/1.73 m^2^	55.2 ± 32.4
Sodium, mEq/L	139 ± 4.00
Potassium, mEq/L	4.20 ± 0.600
B-type natriuretic peptide (BNP), pg/mL	69.0 ± 32.17
Systolic blood pressure, mmHg	125 ± 29.0
Diastolic blood pressure, mmHg	70.0 ± 17.0
LV end-diastolic diameter, mm	45.6 ± 10.4
LV ejection fraction (LVEF), %	60.8 ± 14.0
LVOT velocity-time integral (VTI), cm	19.0 ± 7.22
Left atrial volume index (LAVI), mL/m^2^	42.3 ± 25.5
*E*/*e*′ ratio	12.5 ± 9.01
Stroke volume index (SVI), mL/m^2^	42.1 ± 17.0
TR pressure gradient (TRPG), mmHg	22.0 ± 11.0

Summary of age, sex, anthropometric measures, laboratory results, blood pressure, and echocardiographic parameters. Values are shown as mean ± standard deviation or *n* (%), as appropriate. ECG, electrocardiogram; eGFR, estimated glomerular filtration rate; LV, left ventricle; LVOT, left ventricular outflow tract: TR, tricuspid regurgitation.

The ECG–CXR paired data were randomly split into training (70%), validation (15%), and test (15%) sets (see *Figure 2*). Echocardiographic and biochemical test results associated with these patients were used as ground truth labels for the prediction tasks. Biochemical test results obtained on the same day as the paired ECG and CXR examinations were used. For echocardiographic parameters, the results obtained closest to the ECG/CXR examination date within a 14-day window before or after the examination were used. The median interval between the ECG/CXR examinations and LVEF measurement was 0 days (IQR, 0–0), whereas that between the ECG/CXR examinations and *E*/*e*′ measurement was 0 days (IQR, 0–1) (see Supplementary material online, *Figure S1*(*A*) and *Table S1*).

**Figure 2:**
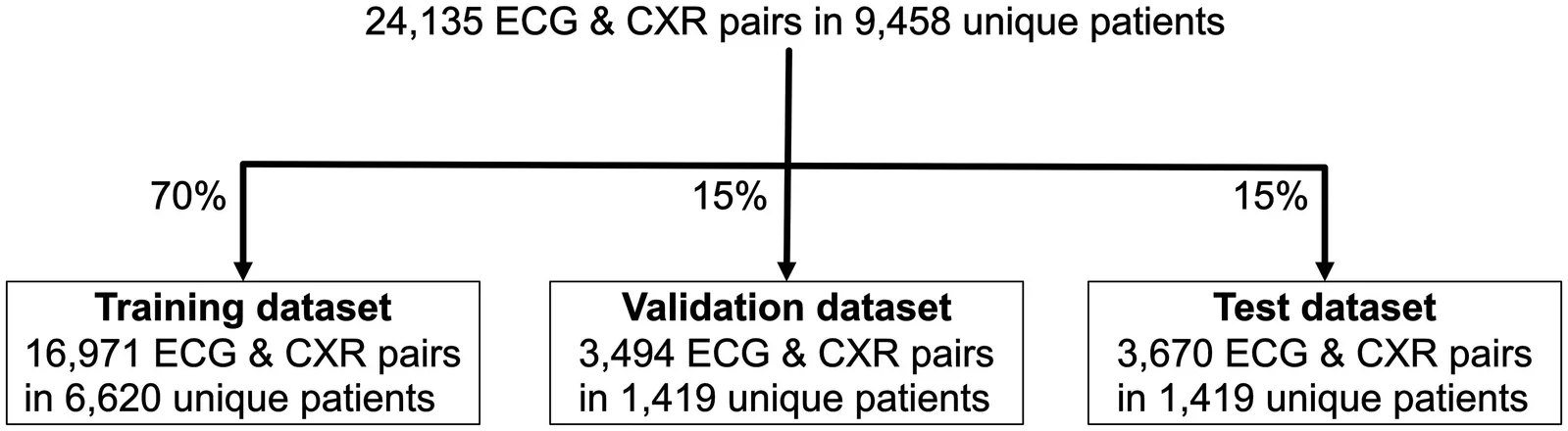
Data splitting. The dataset was split into training, validation, and test sets at a ratio of 70, 15, and 15%, respectively.

### Data pre-processing

ECG data were recorded over 10 s with a sampling interval of 0.002 s (500 Hz) and a voltage resolution of 1.25 μV, yielding 5000 samples per lead. Each recording was stored as a single CSV file. For data augmentation, a continuous 2-second segment (1000 samples) was randomly extracted from each 10-second recording. The data were represented as a floating-point matrix of size 12 × 1000 (leads × time points), and baseline correction was applied by zero-centring during model training.

Chest X-ray images were acquired as frontal greyscale JPEG images with an original resolution exceeding 2000 pixels on each side. For model input, images were resized to 1 × 224 × 224 pixels and normalized with a mean of 0.5 and a standard deviation of 0.5. During training, data augmentation techniques including random cropping, random rotation, and random erasing were applied.^30,31^

### Model architecture

In this study, we developed a two-stage sequential diagnostic model using multimodal data derived from paired ECG and CXR inputs (*Figure 1*). These data were acquired from the same patient on the same day and are therefore assumed to reflect a shared underlying pathophysiological state.

By extracting modality-invariant pathological representations across ECG and CXR, the model enables consistent assessment of disease states as modalities are provided sequentially. In this study, ‘sequential’ refers to the ordered computational updating of predictions as modalities are provided and does not imply that the actual clinical acquisition order or elapsed time between examinations was observed.

#### Step 1: embedding step (CardioCLIP)

In Step 1, we constructed modality-specific encoders to embed ECG and CXR data into a shared pathological representation space based on the CLIP framework^18^ (*Figure 3*). In this stage, ECGs and CXRs acquired from the same patient on the same day were input into their respective encoders, and the model was trained to maximize the cosine similarity between the resulting feature vectors.

**Figure 3:**
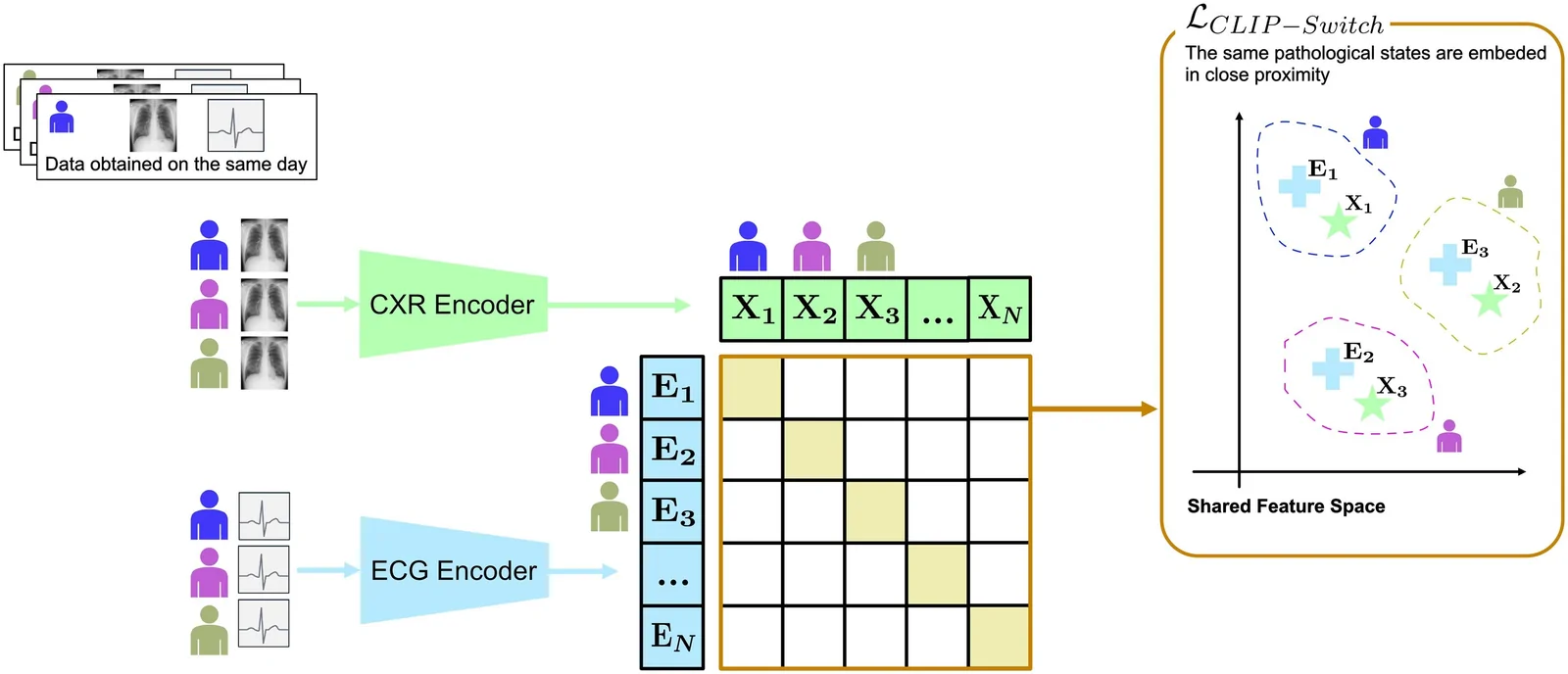
Training of CardioCLIP. Electrocardiogram and chest X-ray data acquired on the same day from the same patient are used to train the electrocardiogram and chest X-ray encoders to embed both modalities into a shared disease space, optimized using the contrastive loss $L_{\mathrm{CLIP}-\mathrm{Switch}}$.

For the ECG encoder, we adopted a previously proposed 1D ResNet-Transformer–based architecture,^24^ while a ViT-B/16 model^32^ was used as the CXR encoder. The CXR encoder was initialized with weights pre-trained on ImageNet-1 K.^33^ The output feature vectors were L2-normalized and trained using a symmetric softmax loss^18^ based on pairwise similarities.

Specifically, for a batch of size *N* consisting of paired ECG and CXR data, let $z_{\mathrm{ECG}}^{i}$ and $z_{\mathrm{CXR}}^{i}$ denote the embedding vectors obtained from the ECG and CXR encoders, respectively, for patient *i*, whose ECG and CXR were acquired on the same calendar day. The loss function $L_{\mathrm{CLIP}}$ is defined as follow:


$$L_{\mathrm{ECG}\to \mathrm{CXR}}=-\frac{1}{N}\overset{N}{\underset{i=1}{\sum }}\frac{\exp(\mathrm{sim}(z_{\mathrm{ECG}}^{i},z_{\mathrm{CXR}}^{i})/\tau )}{\sum_{\;j=1}^{N}\;\exp(\mathrm{sim}(z_{\mathrm{ECG}}^{i},z_{\mathrm{CXR}}^{j})/\tau )}$$

$$L_{\mathrm{CXR}\to \mathrm{ECG}}=-\frac{1}{N}\overset{N}{\underset{i=1}{\sum }}\frac{\exp(\mathrm{sim}(z_{\mathrm{CXR}}^{i},z_{\mathrm{ECG}}^{i})/\tau )}{\sum_{\;j=1}^{N}\;\exp(\mathrm{sim}(z_{\mathrm{CXR}}^{i},z_{\mathrm{ECG}}^{j})/\tau )}$$

$$L_{\mathrm{CLIP}}=L_{\mathrm{ECG}\to \mathrm{CXR}}+L_{\mathrm{CXR}\to \mathrm{ECG}}$$

The embedding vectors *z* are assumed to be normalized, and the similarity function is defined as the dot product, $\mathrm{sim}(z_{\mathrm{ECG}},z_{\mathrm{CXR}})={z_{\mathrm{ECG}}}^{⊤}z_{\mathrm{CXR}}$. The temperature parameter *τ* follows the formulation proposed in CLIP.^18^

In conventional CLIP, embedding vectors tend to form modality-specific clusters in the representation space.^34^ To address this issue, several methods have been proposed to reduce inter-modality discrepancies.^35,36^ However, while conventional CLIP is designed for natural images and language, our target modalities—CXR and ECG—have more constrained feature spaces, which increases the tendency for cluster formation within each modality.

Therefore, existing approaches^35,36^ are not directly applicable in our setting. To mitigate this issue, we introduce a stronger regularization strategy based on a Switch loss, which aims to prevent modality-specific clustering. In the Switch loss, pseudo-negative pairs are generated by swapping modalities within a batch, enabling more robust learning of shared pathological representations. The detailed formulation of the Switch loss is provided in the Supplementary material. The resulting loss function is denoted as $L_{\mathrm{CLIP}-\mathrm{Switch}}$.

#### Step 2: sequential prediction step

In the second stage, a Predictor constructed using a GRU^28^ was trained to sequentially perform binary classification of clinical variables such as LVEF, *E*/*e*′, and BNP, using the pathological embedding vectors obtained in Step 1 as inputs. The Predictor consists of a GRU that updates its hidden state based on the input vectors, followed by a linear layer that performs binary classification from the GRU hidden state. Details of the training procedure are described in the Supplementary material.

Based on the trained Predictor and the encoders learned in Step 1, we describe below the process of sequentially estimating the probability of abnormal laboratory values for a given patient *i*, assuming that the modalities are provided to the model in the order ECG followed by CXR.

The GRU outputs a hidden state $h_{t}$ given the previous hidden state $h_{t-1}$ and an input vector *z*. Let $z_{\mathrm{ECG}}^{i}$ denote the embedding vector obtained from the ECG of patient *i*. The hidden state after processing the ECG is computed as:


$$h_{\mathrm{ECG}}=\mathrm{GRU}(z_{\mathrm{ECG}}^{i},h_{\mathrm{prior}})$$

where the initial prior hidden state $h_{\mathrm{prior}}$ is initialized as a zero vector. The predicted probability at this stage, $p_{\mathrm{ECG}}$, is obtained using a linear binary classifier as


$$p_{\mathrm{ECG}}=\mathrm{Linear}(h_{\mathrm{ECG}})$$

Subsequently, when the CXR of the same patient becomes available, the final prediction probability $p_{\mathrm{ECG}\to \mathrm{CXR}}$ is computed as:


$$h_{\mathrm{ECG}\to \mathrm{CXR}}=\mathrm{GRU}(z_{\mathrm{ECG}}^{i},h_{\mathrm{ECG}})$$

$$p_{\mathrm{ECG}\to \mathrm{CXR}}=\mathrm{Linear}(h_{\mathrm{ECG}\to \mathrm{CXR}})$$

Using this approach, an initial estimate of abnormality can be obtained at the stage of $p_{\mathrm{ECG}}$, enabling an intermediate prediction before the second modality is incorporated. The final prediction $p_{\mathrm{ECG}\to \mathrm{CXR}}$ represents a multimodal estimate based on both ECG and CXR information. Thus, this framework enables sequential updating and multimodal prediction of abnormal laboratory values. The same procedure applies when the modalities are provided to the model in the reverse order (CXR followed by ECG), and the framework can be naturally extended to an arbitrary number of examinations.

### Assessment of echocardiographic abnormalities and natriuretic peptide elevation

The Forrester classification^37^ is widely used for haemodynamic assessment in heart failure; however, it requires invasive catheterization to measure cardiac index and pulmonary capillary wedge pressure. In the present retrospective dataset, direct invasive haemodynamic parameters were not available. Therefore, non-invasive surrogate markers are commonly used. Stroke volume index (SVI) and *E*/*e*′ have been proposed as surrogates for cardiac index and pulmonary capillary wedge pressure, respectively.^38^ However, due to limited reproducibility of SVI, we used LVEF, an alternative indicator of left ventricular systolic function, in place of SVI. In addition, BNP is a well-established biomarker for assessing heart failure severity and prognosis.^39,40^

Accordingly, we evaluated echocardiographic abnormalities and natriuretic peptide elevation by predicting abnormalities in LVEF, *E*/*e*′, and BNP. ECG–CXR paired data with corresponding labels for these indicators were used to obtain CardioCLIP embeddings, which were then input to the Step 2 sequential prediction model to estimate the probability of abnormality in a binary classification setting. Abnormal values were defined as LVEF < 50%, *E*/*e*′> 15, and BNP > 100 pg/mL. The validity of these thresholds is described in the Supplementary material. These endpoints should be interpreted as clinically relevant surrogate markers rather than direct invasive haemodynamic measurements.

### Evaluation on additional clinical variables

In addition to these primary surrogate endpoints, we evaluated model performance across a broader range of clinical variables. Thresholds for each variable are summarized in *Table 2*, and their clinical validity is described in the Supplementary material.

**Table 2:** Comparison of multimodal and unimodal models

Indicator	Comparison	Lower 95% CI	Upper 95% CI	Bootstrap proportion (ΔAUC ≤ 0)
BNP > 100	**ECG → CXR vs. ECG**	0.00423	0.0221	0.002
	**ECG → CXR vs. CXR**	0.0397	0.0616	<0.001
	CXR → ECG vs. ECG	−0.00016	0.0191	0.027
	**CXR → ECG vs. CXR**	0.0369	0.0568	<0.001
LVEF < 50	**ECG → CXR vs. ECG**	0.0181	0.0881	0.001
	**ECG → CXR vs. CXR**	0.0188	0.0763	<0.001
	**CXR → ECG vs. ECG**	0.0190	0.0801	<0.001
	**CXR → ECG vs. CXR**	0.0111	0.0751	0.004
*E*/*e*′ > 15	ECG → CXR vs. ECG	−0.0199	0.0375	0.304
	**ECG → CXR vs. CXR**	0.0197	0.0671	<0.001
	CXR → ECG vs. ECG	−0.0213	0.0353	0.331
	**CXR → ECG vs. CXR**	0.0175	0.0659	<0.001
Subsequent severe BNP elevation	ECG → CXR vs. ECG	−0.00306	0.0439	0.043
	**ECG → CXR vs. CXR**	0.00796	0.0533	0.004
	**CXR → ECG vs. ECG**	0.00159	0.0489	0.019
	**CXR → ECG vs. CXR**	0.0129	0.0579	0.004
AR ≥ moderate	**ECG → CXR vs. ECG**	0.0357	0.1079	<0.001
	ECG → CXR vs. CXR	−0.0427	0.0286	0.662
	**CXR → ECG vs. ECG**	0.0301	0.0969	<0.001
	CXR → ECG vs. CXR	−0.0548	0.0226	0.799
AS ≥ moderate	ECG → CXR vs. ECG	−0.0149	0.0507	0.163
	ECG → CXR vs. CXR	−0.0123	0.0826	0.058
	CXR → ECG vs. ECG	−0.0100	0.0508	0.111
	CXR → ECG vs. CXR	−0.0115	0.0883	0.063
DBP > 90	ECG → CXR vs. ECG	−0.0615	0.0483	0.622
	ECG → CXR vs. CXR	−0.0423	0.0932	0.205
	CXR → ECG vs. ECG	−0.0958	0.0630	0.703
	CXR → ECG vs. CXR	−0.0283	0.0573	0.260
SBP > 140	ECG → CXR vs. ECG	−0.0518	0.0282	0.684
	**ECG → CXR vs. CXR**	0.0191	0.0932	0.006
	CXR → ECG vs. ECG	−0.0558	0.0290	0.716
	**CXR → ECG vs. CXR**	0.0223	0.0841	0.001
eGFR < 60	**ECG → CXR vs. ECG**	0.0150	0.0409	<0.001
	**ECG → CXR vs. CXR**	0.00357	0.0298	0.010
	**CXR → ECG vs. ECG**	0.0135	0.0410	<0.001
	**CXR → ECG vs. CXR**	0.00379	0.0270	0.006
HbA1c ≥ 6.5	**ECG → CXR vs. ECG**	0.0290	0.0784	<0.001
	ECG → CXR vs. CXR	−0.00392	0.0442	0.058
	**CXR → ECG vs. ECG**	0.0360	0.0823	<0.001
	**CXR → ECG vs. CXR**	0.000784	0.0499	0.023
Hb < 12	**ECG → CXR vs. ECG**	0.0282	0.0608	<0.001
	ECG → CXR vs. CXR	−0.00254	0.0283	0.046
	**CXR → ECG vs. ECG**	0.0275	0.0566	<0.001
	CXR → ECG vs. CXR	−0.00708	0.0265	0.113
K > 5.5	**ECG → CXR vs. ECG**	0.0356	0.199	0.001
	ECG → CXR vs. CXR	−0.132	0.0590	0.787
	**CXR → ECG vs. ECG**	0.00186	0.205	0.023
	CXR → ECG vs. CXR	−0.169	0.0386	0.885
Na > 145	**ECG → CXR vs. ECG**	0.0426	0.344	0.010
	ECG → CXR vs. CXR	−0.145	0.0507	0.898
	**CXR → ECG vs. ECG**	0.0647	0.403	0.010
	CXR → ECG vs. CXR	−0.0950	0.0553	0.721
LAVI > 34	ECG → CXR vs. ECG	−0.0217	0.0356	0.310
	**ECG → CXR vs. CXR**	0.00691	0.0690	0.010
	CXR → ECG vs. ECG	−0.0202	0.0349	0.319
	**CXR → ECG vs. CXR**	0.00371	0.0710	0.011
K < 3.4	ECG → CXR vs. ECG	−0.000235	0.118	0.027
	ECG → CXR vs. CXR	−0.0740	0.0620	0.595
	CXR → ECG vs. ECG	−0.0400	0.0852	0.249
	CXR → ECG vs. CXR	−0.0927	0.0177	0.912
Na < 135	ECG → CXR vs. ECG	−0.0119	0.0539	0.101
	ECG → CXR vs. CXR	−0.0462	0.0401	0.584
	**CXR → ECG vs. ECG**	0.00168	0.0729	0.019
	CXR → ECG vs. CXR	−0.0281	0.0536	0.277
LVDd > 55	**ECG → CXR vs. ECG**	0.0201	0.0803	<0.001
	**ECG → CXR vs. CXR**	0.00149	0.0515	0.021
	**CXR → ECG vs. ECG**	0.0170	0.0768	<0.001
	CXR → ECG vs. CXR	−0.00206	0.0483	0.033
LVOT VTI < 15	ECG → CXR vs. ECG	−0.0358	0.0354	0.499
	**ECG → CXR vs. CXR**	0.00685	0.0954	0.013
	CXR → ECG vs. ECG	−0.0414	0.0267	0.657
	CXR → ECG vs. CXR	−0.00355	0.0909	0.031
MR ≥ moderate	ECG → CXR vs. ECG	−0.00795	0.0591	0.074
	**ECG → CXR vs. CXR**	0.000126	0.0247	0.025
	**CXR → ECG vs. ECG**	0.00715	0.0354	0.003
	CXR → ECG vs. CXR	−0.0221	0.0393	0.313
BMI > 25	**ECG → CXR vs. ECG**	0.0209	0.0785	<0.001
	ECG → CXR vs. CXR	−0.0184	0.0141	0.632
	**CXR → ECG vs. ECG**	0.0163	0.0723	<0.001
	CXR → ECG vs. CXR	−0.0283	0.00911	0.831
SVI < 35	ECG → CXR vs. ECG	−0.0353	0.0329	0.511
	**ECG → CXR vs. CXR**	0.0252	0.101	<0.001
	CXR → ECG vs. ECG	−0.0317	0.0313	0.495
	**CXR → ECG vs. CXR**	0.0242	0.105	0.002
TRPG > 30	ECG → CXR vs. ECG	−0.0318	0.0241	0.635
	**ECG → CXR vs. CXR**	0.0468	0.115	<0.001
	CXR → ECG vs. ECG	−0.0255	0.0229	0.540
	**CXR → ECG vs. CXR**	0.0474	0.124	<0.001
BUN > 20	ECG → CXR vs. ECG	0.0128	0.0481	0.001
	ECG → CXR vs. CXR	0.00155	0.0265	0.013
	CXR → ECG vs. ECG	0.0179	0.0518	<0.001
	CXR → ECG vs. CXR	0.00761	0.0290	0.001

For each clinical indicator, the performance of the multimodal models (ECG → CXR and CXR → ECG) was compared with that of the unimodal models (electrocardiogram and CXR) by calculating differences in the area under the receiver operating characteristic curve (ΔAUC) using paired bootstrap resampling. Two-sided percentile-based 95% confidence intervals are reported, and statistical significance was determined exclusively by whether the 95% confidence interval excluded zero. One-sided empirical bootstrap tail probabilities, defined as the proportion of bootstrap samples with ΔAUC ≤ 0, are also reported but were not used to determine statistical significance. Statistically significant differences are highlighted in bold.

### External validation using the MIMIC-IV dataset

To assess generalizability, external validation was conducted using the MIMIC dataset.^41–44^ MIMIC-IV,^41^ which provides laboratory data, along with MIMIC-IV-CXR^42^ and MIMIC-IV-ECG,^44^ includes patient identifiers and examination timestamps. Using these identifiers, we extracted paired ECG, CXR, and laboratory data acquired from the same patient on the same date.

Using this dataset, we performed binary classification for N-terminal pro-BNP (NT-proBNP) as a surrogate marker for BNP. A model trained on our institutional dataset was evaluated on the MIMIC-IV dataset for abnormal NT-proBNP prediction. The external validation cohort included 40 285 patients and 94 105 samples. The median NT-proBNP level was 1249 pg/mL (interquartile range: 294–4177 pg/mL).

### Model evaluation and statistical analysis

Model performance for binary classification was evaluated using the area under the receiver operating characteristic curve (ROC–AUC). For each task, including LVEF prediction, the mean AUC and 95% confidence intervals were estimated using bootstrap resampling with 1000 iterations.

For model comparisons, paired bootstrap resampling (1000 iterations) was used. In each iteration, the difference in AUC (ΔAUC) was calculated using the same resampled test set for both models. We report two-sided percentile-based 95% confidence intervals and one-sided empirical bootstrap tail probabilities, defined as the proportion of bootstrap samples with ΔAUC ≤ 0. Statistical significance was determined exclusively by whether the 95% confidence interval excluded zero. All analyses were performed using Python 3.8 and PyTorch 2.1.

## Results

### Patient characteristics

This study included 9458 patients who visited the Department of Cardiology at Kobe University Hospital between January 2012 and February 2022 and underwent both ECG and frontal CXR imaging on the same day. The mean age of the study population was 69.6 ± 22.1 years. The mean BNP level was 69.0 ± 32.17 pg/mL, and the mean LVEF was 60.8 ± 14.0%.

As shown in *Figure 2*, the entire cohort was randomly divided into three datasets: 6620 patients (70%) for training, 1419 (15%) for validation, and 1419 (15%) for testing (*Table 1*).

### Learning and evaluation of the pathological representation space with CardioCLIP

To embed ECG and CXR into a shared pathological representation space, CardioCLIP was trained using a modified contrastive loss, $L_{\mathrm{CLIP}-\mathrm{Switch}}$, as illustrated in *Figure 3*. We assumed that ECG and CXR data acquired from the same patient at the same time point reflect the same underlying pathological state, and trained modality-specific encoders (ECG and CXR encoders) to learn this shared representation space.

To evaluate the effectiveness of the learned representations, we performed a cross-modal retrieval task. Given an ECG from a patient, we retrieved the most similar CXR within the same batch and measured the proportion of cases in which the retrieved image corresponded to the same patient and acquisition time (Top-1 accuracy). Similarly, we evaluated retrieval performance using CXR as the query and ECG as the target modality. The average of these two Top-1 accuracies was used as the performance metric.

The results are shown in *Figure 4*. Although the accuracy decreased with increasing batch size, the embeddings learned by CardioCLIP maintained a Top-1 accuracy of nearly 40% even at a batch size of 30, indicating that the pathological representation space was effectively learned.

**Figure 4:**
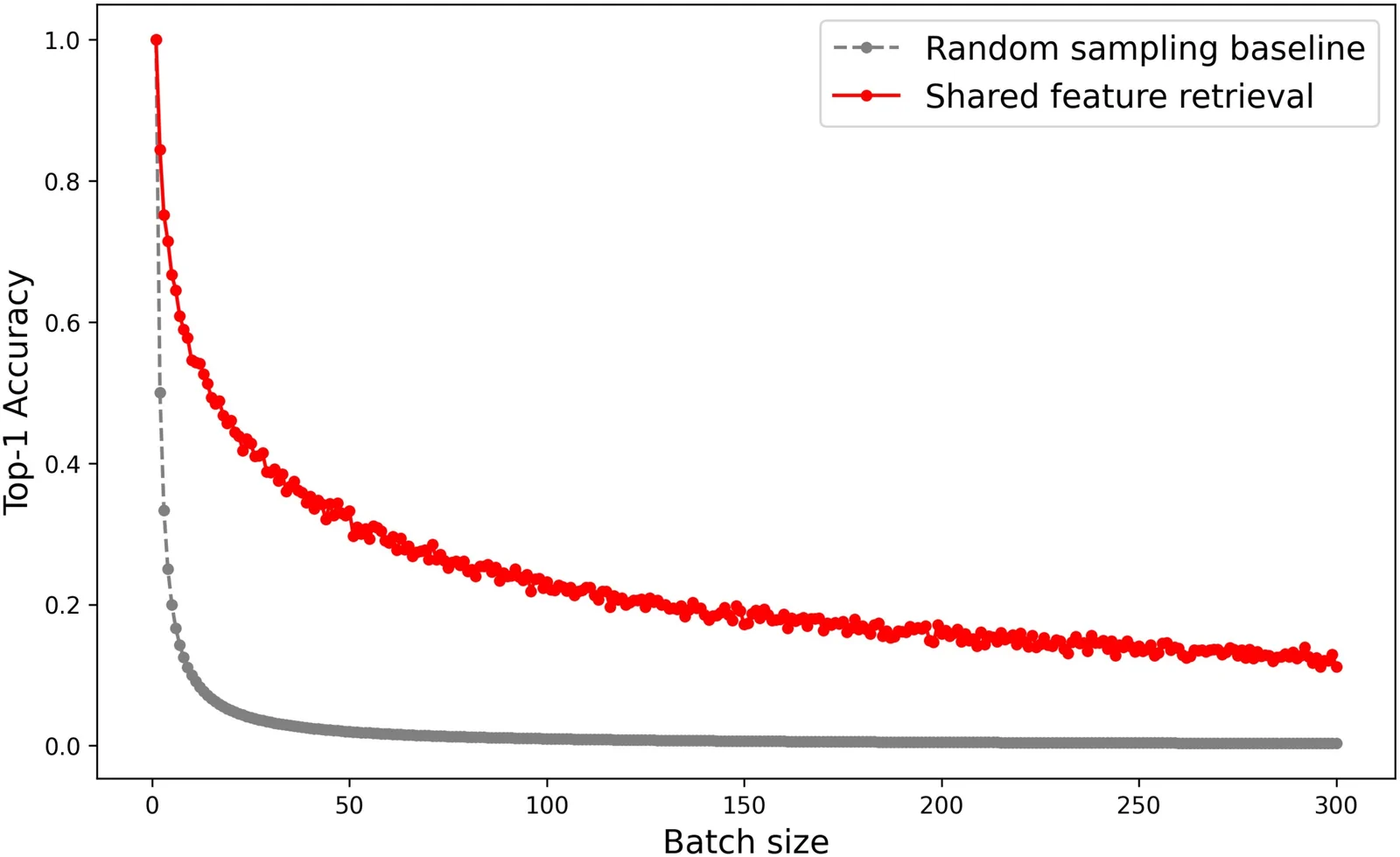
Cross-modal retrieval performance of CardioCLIP across batch sizes. For a random batch, the Top-1 accuracy of retrieving the corresponding chest X-ray from an electrocardiogram of the same patient and, conversely, retrieving the corresponding electrocardiogram from a chest X-ray was computed. The mean of these bidirectional Top-1 accuracies is plotted for each batch size.

### Evaluation of echocardiographic abnormalities and natriuretic peptide elevation

As a primary outcome of this study, we evaluated the performance of the proposed method in predicting echocardiographic abnormalities and natriuretic peptide elevation, including BNP, LVEF, and *E*/*e*′, using binary classification. Predictive performance was assessed using the AUC–ROC curve. Receiver operating characteristic curves for each indicator are shown in *Figure 5(A)*.

**Figure 5:**
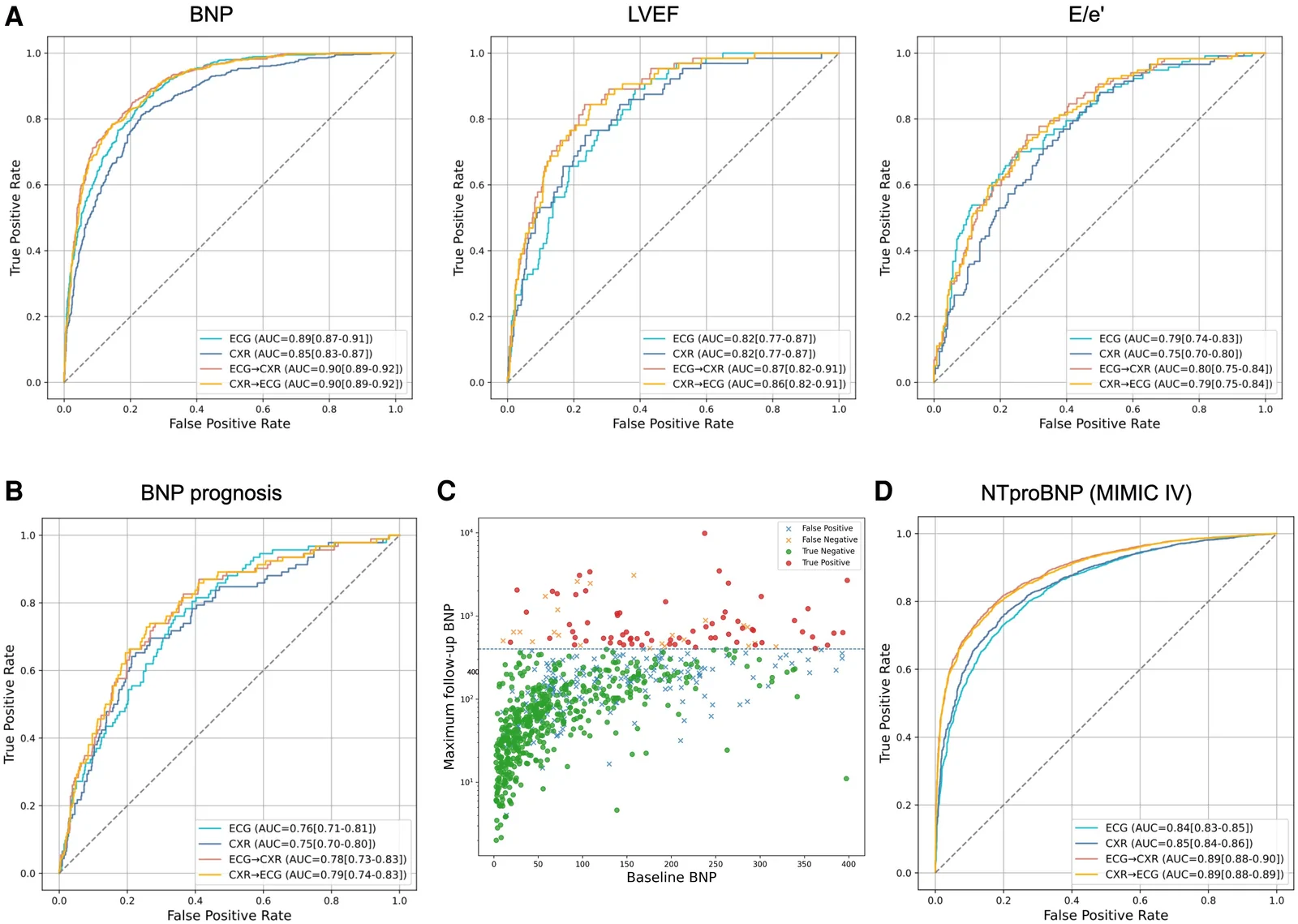
Receiver operating characteristic analyses. (*A*) Receiver operating characteristic curves for the prediction of B-type natriuretic peptide, left ventricular ejection fraction, and *E*/*e*′ abnormalities. (*B*) Receiver operating characteristic curve for prediction of subsequent severe B-type natriuretic peptide elevation. (*C*) Scatter plot for subsequent severe B-type natriuretic peptide elevation, with baseline B-type natriuretic peptide on the *x*-axis and the maximum B-type natriuretic peptide value measured within 3 years after the baseline measurement in the same patient on the *y*-axis. Data points shown as circles indicate cases in which the model correctly classified subsequent severe B-type natriuretic peptide elevation, whereas crosses indicate cases with incorrect predictions. (*D*) Receiver operating characteristic curve for external validation of NT-proBNP prediction in the MIMIC-IV dataset.

For BNP > 100 pg/mL, the AUC was 0.89 for the ECG model and 0.85 for the CXR model, indicating superior performance of the ECG-based model. The multimodal model further improved performance, achieving an AUC of 0.90 when inputs were provided in the order ECG → CXR and 0.90 for CXR → ECG. Bootstrap analysis demonstrated that the ECG → CXR model significantly outperformed both unimodal models, whereas the CXR → ECG model significantly outperformed the CXR model but not the ECG model (*Table 2*).

For LVEF < 50%, the AUC was 0.82 for both ECG and CXR models. The multimodal models improved performance to 0.87 (ECG → CXR) and 0.86 (CXR → ECG), both of which were significantly higher than the unimodal models (*Table 2*). Threshold-dependent performance metrics, including sensitivity and specificity, are presented in Supplementary material online, *Table S2*. For the prediction of LVEF < 50%, the ECG → CXR and CXR → ECG models achieved the highest sensitivity of 0.789.

For *E*/*e*′ > 15, the AUC was 0.79 for the ECG model and 0.75 for the CXR model. The multimodal models achieved AUCs of 0.80 (ECG → CXR) and 0.79 (CXR → ECG). While both multimodal models significantly outperformed the CXR-only model, no significant difference was observed compared with the ECG-only model (*Table 2*).

To evaluate prognostic performance, we performed a binary classification task among patients with BNP <400 pg/mL at baseline. Follow-up BNP measurements obtained after the baseline date and within 3 years were identified, with measurements obtained on the baseline date excluded. The highest BNP value recorded during this follow-up period was used as the target, and subsequent severe BNP elevation was defined as a maximum follow-up BNP level >400 pg/mL. For patients who died within 3 years, only BNP measurements obtained before death were considered. Patients without any eligible follow-up BNP measurement during the 3-year period or before death were excluded from this analysis. The corresponding ROC curves are shown in *Figure 5(B)*. The AUCs for the ECG, CXR, ECG → CXR, and CXR → ECG models were 0.76, 0.75, 0.78, and 0.79, respectively.

To further assess prediction of subsequent severe BNP elevation in patients with low baseline BNP, *Figure 5(C)* plots baseline BNP against the maximum BNP within 3 years. Classification based on the optimal threshold defined by Youden’s index^45^ using the CXR → ECG model is shown, where circles indicate correctly classified cases and crosses indicate misclassified cases. These results show discrimination for subsequent severe BNP elevation across a wide range of baseline BNP values (0–400 pg/mL).

### Performance of the sequential model across multiple clinical variables

To assess the generalizability of the embeddings obtained by CardioCLIP, we performed binary classification across a range of clinical variables and calculated the AUC for each outcome. The results are shown in *Figure 6*.

**Figure 6:**
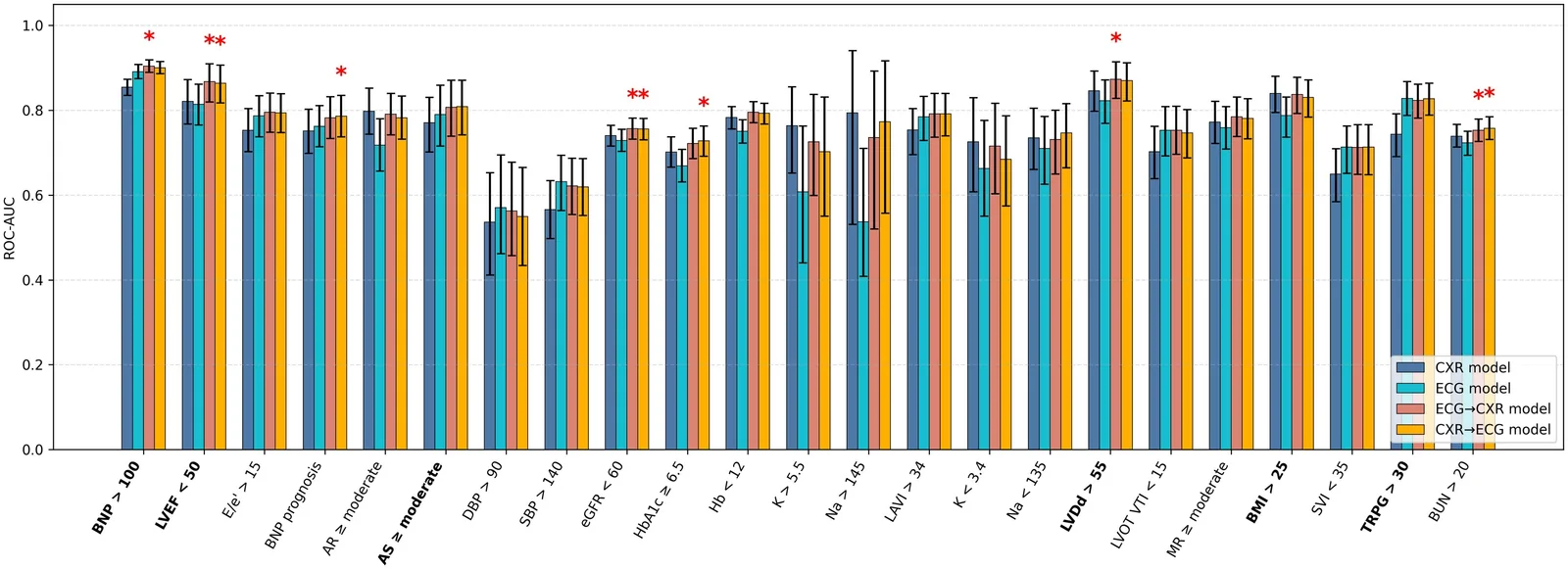
AUC across multiple laboratory measurements. For each laboratory indicator, an asterisk (*) indicates models in which either multimodal approach (ECG → CXR or CXR → ECG) showed a statistically significant difference in AUC compared with both unimodal models (electrocardiogram and chest X-ray). Indicators with an AUC >0.8 are shown in bold.

For each variable, statistical significance was evaluated by comparing AUC differences between the multimodal model and the two unimodal models (ECG and CXR). Models with statistically significant differences are indicated with an asterisk. Variables with an AUC >0.8 are highlighted in bold.

These results demonstrate that the model achieves high predictive performance, particularly for clinically relevant surrogate cardiovascular endpoints. For example, in BNP and LVEF prediction, the multimodal models improved discrimination over the unimodal models for selected comparisons, indicating effective integration of complementary information from ECG and CXR by CardioCLIP. In contrast, performance was lower for variables such as electrolyte levels, which are inherently difficult to infer from ECG or CXR data alone.

### External validation using the MIMIC-IV dataset

To evaluate the generalizability of CardioCLIP, we conducted external validation using the MIMIC-IV dataset with models trained on our institutional dataset. Although MIMIC-IV includes echocardiographic data, corresponding measurements such as LVEF and *E*/*e*′ were not consistently available.^46^ Therefore, we evaluated performance using NT-proBNP as a surrogate for BNP.

The results are shown in *Figure 5(D)*. The AUCs of the ECG and CXR models were 0.84 and 0.85, respectively, which were slightly lower but comparable to those observed in our internal test dataset. The multimodal models further improved performance, achieving AUCs of 0.89 for both ECG → CXR and CXR → ECG input orders, consistent with the trends observed in the internal evaluation.

## Discussion

In this single-centre study of 9458 patients and 24 135 same-day ECG–CXR pairs, we developed CardioCLIP, a sequential multimodal framework that learns a shared pathological representation from ECG and CXR and supports inference regardless of modality order. CardioCLIP achieved AUCs of 0.90 for BNP >100 pg/mL, 0.87 for LVEF <50%, and 0.80 for *E*/*e*′ > 15, and showed discrimination for subsequent severe BNP elevation within 3 years with an AUC of 0.79. In external validation using 40 285 patients from MIMIC-IV, the multimodal model maintained an AUC of 0.89 for abnormal NT-proBNP prediction, supporting the potential transportability of the learned representation.

### Leveraging a shared pathological representation space

The learned representation space demonstrated strong cross-modal alignment, as evidenced by high retrieval accuracy in both ECG-to-CXR and CXR-to-ECG tasks (*Figure 4*). Although direct prediction of CXR findings from ECG is inherently challenging, prior studies have shown that such cross-modal inference is feasible using machine learning.^47^ This suggests that CardioCLIP successfully captures shared pathological features across modalities. Importantly, the learned embeddings can be applied to a wide range of downstream tasks.

### Application to echocardiographic abnormalities and natriuretic peptide elevation

In predicting echocardiographic abnormalities and natriuretic peptide elevation (BNP > 100 pg/mL, LVEF < 50%, and *E*/*e*′ > 15), CardioCLIP achieved AUCs of 0.90, 0.87, and 0.80, respectively, using non-invasive and routinely available modalities (*Figure 5A*). These results indicate that the model extracts clinically meaningful features from ECG and CXR. Furthermore, the multimodal model improved discrimination for selected endpoints and comparisons relative to the unimodal models.

This framework performs ordered computational updating as information from multiple tests is provided sequentially. As such, CardioCLIP may provide decision support for identifying patients with abnormal echocardiographic indices or natriuretic peptide elevation during heart failure-related evaluation.

Unlike conventional multimodal models,^7–11^ the proposed framework supports unimodal inference and both evaluated modality-input orders. While prior studies have demonstrated prediction of cardiac surrogate endpoints or prognosis from ECG and CXR,^10,11^ few have incorporated a shared pathological representation with sequential inference capability. Although direct comparison with previous approaches is challenging, prior work using multimodal data including ECG reported AUCs of ∼0.9 for NT-proBNP prediction in the MIMIC-IV dataset.^9^ Our method achieved comparable performance while maintaining flexibility in modality usage, as demonstrated by external validation (*Figure 5D*).

In addition, CardioCLIP showed discrimination, with an AUC of 0.79, for subsequent severe BNP elevation (BNP > 400 pg/mL) (*Figure 5B*). This finding is limited to the biomarker endpoint and should not be interpreted as direct evidence of future heart failure progression.

BNP/NT-proBNP prediction was retained as a major endpoint because natriuretic peptides are guideline-supported biomarkers for heart failure diagnosis, risk stratification, and prognosis, and elevated values can prompt further evaluation, closer follow-up, or additional testing.^39,40^ In the present framework, prediction of BNP or NT-proBNP elevation from ECG and CXR is not intended to replace biomarker testing. Rather, it may identify patients whose routinely acquired tests contain patterns consistent with cardiac stress and who may benefit from natriuretic peptide measurement, echocardiography, or closer clinical assessment. Nevertheless, natriuretic peptide concentrations are influenced by both cardiac and non-cardiac factors, including age, renal function, atrial fibrillation, obesity, and acute illness.^40^ Therefore, model outputs should be interpreted as probabilistic risk estimates rather than definitive diagnostic labels.

In contrast, performance was lower for variables not directly related to the primary cardiac endpoints (*Figure 6*). For example, electrolyte levels such as sodium showed limited predictability, likely due to their weak association with ECG or CXR features. Although potassium levels can influence ECG findings, predictive performance remained modest, possibly due to class imbalance and limited positive samples. Overall, ECG-based models tended to outperform CXR-based models, which may reflect the richer temporal and physiological information contained in ECG signals compared with the primarily structural information captured by CXR. Conversely, for variables such as body mass index (BMI), which reflect physical characteristics, CXR-based predictions outperformed ECG-based models.

### Clinical implications of the sequential prediction model

The proposed sequential prediction framework is intended to support prioritization for additional evaluation among patients in whom ECG and CXR have already been obtained as part of routine care. It may estimate the risk of echocardiographic abnormalities or natriuretic peptide elevation but is not intended to replace echocardiography, BNP/NT-proBNP measurement, or physician judgment. Whether this approach reduces testing, improves workflow, or improves outcomes should be evaluated in future prospective and reader studies.

By enabling prediction of echocardiographic and laboratory abnormalities from rapid and non-invasive tests such as ECG and CXR, CardioCLIP may be particularly useful in resource-limited settings, including outpatient clinics and emergency departments.

Furthermore, while echocardiography requires specialized expertise and may have limited reproducibility, the proposed model achieved high performance in predicting echocardiographic indices such as LVEF and *E*/*e*′. Therefore, CardioCLIP may provide a practical and stable adjunct for screening echocardiographic abnormalities. Given that many clinical variables, including BNP, LVEF, and *E*/*e*′, achieved AUCs around 0.8, the proposed framework demonstrates potential as a decision-support approach that requires prospective validation before clinical implementation.

### Limitations

This study has several limitations. First, in multimodal learning, irrelevant modality-specific information may act as noise and degrade performance. Although the multimodal model significantly improved performance for primary surrogate endpoints, this issue may still persist.

Second, contrastive learning frameworks such as CLIP primarily capture shared information across modalities, potentially overlooking modality-specific features that may also be clinically relevant.

Third, there are limitations related to the dataset. Left ventricular ejection fraction and *E*/*e*′ were used as surrogate measures to approximate the Forrester classification; however, direct measurements of cardiac index and pulmonary capillary wedge pressure were not available for validation. B-type natriuretic peptide and NT-proBNP levels can also be influenced by age, renal function, atrial fibrillation, obesity, and acute illness. Although the ECG and CXR examinations were performed on the same day, the exact acquisition time of the CXR images was not recorded. Therefore, the time interval between ECG and CXR acquisition within the same day could not be determined. This missing timing information does not affect the computational definition of sequential updating; however, same-day acquisition does not establish that the examinations became available at distinct clinical decision points. Consequently, the present study could not evaluate the operational time window in which an intermediate prediction might influence clinical workflow. In addition, this was a single-centre study, and the dataset may contain institutional bias and limited diversity.

Although the present study demonstrates the feasibility and diagnostic performance of a sequential multimodal model, it did not evaluate whether model outputs improve physician decision-making, diagnostic confidence, reading time, or downstream testing decisions. Future prospective reader studies involving clinicians with different levels of experience are needed to determine the clinical utility and workflow impact of AI-assisted interpretation.

Fourth, although the model improved AUC-based discrimination for selected endpoints, the present retrospective analysis does not establish clinical utility, workflow feasibility, or outcome benefit. Clinical implementation would require further evaluation of temporal alignment, outcome-measurement timing, missing-modality handling, workflow integration, data governance, and whether the observed AUC improvement justifies the added complexity of multimodal deployment.

Future work should include validation using multicentre datasets and extension of the framework to incorporate additional modalities, such as cardiac MRI and biomarkers. These efforts may further enhance the generalizability and clinical utility of CardioCLIP.

## Conclusions

In this study, we proposed a sequential multimodal prediction framework that supports unimodal inference and either modality-input order. We developed CardioCLIP, a multimodal representation learning model that embeds ECG and CXR data into a shared pathological representation space.

By representing heterogeneous medical data within a unified space, CardioCLIP enables semantic integration across modalities. This allows efficient and sequential inference using a lightweight downstream predictor based on learned embeddings, in either evaluated modality-input order.

The proposed method showed promising discriminatory performance for echocardiographic abnormalities and natriuretic peptide elevation, including BNP, LVEF, and *E*/*e*′, and may support prioritization for additional evaluation, subject to prospective validation. In addition, the model showed discrimination for subsequent severe BNP elevation (BNP > 400 pg/mL within 3 years) among patients with BNP < 400 pg/mL at baseline. These findings support the feasibility of CardioCLIP as an order-flexible sequential multimodal framework; clinical utility remains to be established prospectively.

Overall, the proposed framework may represent a clinically adaptable approach to disease state prediction, offering a more flexible alternative to conventional machine learning-based diagnostic support systems in cardiovascular medicine, although its clinical utility and workflow impact require prospective validation.

## Supplementary Material

ztag158_Supplementary_Data

## Data Availability

The data that support the findings of this study are derived from two sources. The institutional dataset collected at Kobe University Hospital contains sensitive patient information and is therefore not publicly available. Access to these data may be granted upon reasonable request to the corresponding author and with appropriate approval from the institutional review board and data governance committees, in accordance with local regulations and ethical guidelines. The external validation dataset used in this study was obtained from the MIMIC-IV database. MIMIC-IV is a publicly available database, and access can be obtained after completion of the required training and data use agreement. Details are available at: https://physionet.org/content/mimiciv/. Code for data pre-processing, model training, evaluation is available on GitHub (https://github.com/shbst/CardioCLIP). The repository contains the implementation of the proposed CardioCLIP framework, including the embedding step (contrastive learning) and the sequential prediction step (GRU-based predictor).

## References

[1] McKinney SM, Sieniek M, Godbole V, Godwin J, Antropova N, Ashrafian H, et al International evaluation of an AI system for breast cancer screening. Nature 2020;577:89–94. 10.1038/s41586-019-1799-6 31894144

[2] Liu Y, Jain A, Eng C, Way DH, Lee K, Bui P, et al A deep learning system for differential diagnosis of skin diseases. Nat Med 2020;26:900–908. 10.1038/s41591-020-0842-3 32424212

[3] Bock C, Walter JE, Rieck B, Strebel I, Rumora K, Schaefer I, et al Enhancing the diagnosis of functionally relevant coronary artery disease with machine learning. Nat Commun 2024;15:5034. 10.1038/s41467-024-49390-y PMC11169272 38866791

[4] Ouyang D, He B, Ghorbani A, Yuan N, Ebinger J, Langlotz CP, et al Video-based AI for beat-to-beat assessment of cardiac function. Nature 2020;580:252–256. 10.1038/s41586-020-2145-8 PMC8979576 32269341

[5] Sau A, Pastika L, Sieliwonczyk E, Patlatzoglou K, Ribeiro AH, McGurk KA, et al Artificial intelligence-enabled electrocardiogram for mortality and cardiovascular risk estimation: a model development and validation study. Lancet Digit Health 2024;6:e791–e802. 10.1016/S2589-7500(24)00172-9 39455192

[6] Fahmy AS, Csecs I, Arafati A, Assana S, Yankama TT, Al-Otaibi T, et al An explainable machine learning approach reveals prognostic significance of right ventricular dysfunction in nonischemic cardiomyopathy. JACC Cardiovasc Imaging 2022;15:766–779. 10.1016/j.jcmg.2021.11.029 35033500

[7] Liu PY, Hsing SC, Tsai DJ, Lin C, Lin CS, Wang CH, et al A deep-learning-enabled electrocardiogram and chest X-ray for detecting pulmonary arterial hypertension. J Imaging Inform Med 2025;38:747–756. 10.1007/s10278-024-01225-4 PMC11950589 39136826

[8] Kishikawa R, Kodera S, Setoguchi N, Tanabe K, Kushida S, Nanasato M, et al An ensemble learning model for detection of pulmonary hypertension using electrocardiogram, chest X-ray, and brain natriuretic peptide. Eur Heart J Digit Health 2025;6:209–217. 10.1093/ehjdh/ztae097 PMC11914732 40110214

[9] Alcaraz JML, Strodthoff N. Abnormality prediction and forecasting of laboratory values from electrocardiogram signals using multimodal deep learning. Sci Rep 2025;15:39362. 10.1038/s41598-025-26715-5 PMC12603147 41214165

[10] Liu W-T, Lin C-S, Tsao T-P, Lee C-C, Cheng C-C, Chen J-T, et al A deep-learning algorithm-enhanced system integrating electrocardiograms and chest X-rays for diagnosing aortic dissection. Can J Cardiol 2022;38:160–168. 10.1016/j.cjca.2021.09.028 34619339

[11] Nagai S, Nishimori M, Shinohara M, Tanaka H, Otake H. Multimodal deep learning model for enhanced early detection of aortic stenosis integrating ECG and chest x-ray with cooperative learning. Front Radiol 2025;5:1698680. 10.3389/fradi.2025.1698680 PMC12685832 41378244

[12] Mmformer: multimodal medical transformer for incomplete multimodal learning of brain tumor segmentation. In: Zhang Y, He N, Yang J, Li Y, Wei D, Huang Y, et al, eds. International Conference on Medical Image Computing and Computer-Assisted Intervention. Cham, Switzerland: Springer; 2022:107–117.

[13] Robinet L, Berjaoui A, Moyal EC-J. Multimodal masked autoencoder pre-training for 3D MRI-based brain tumor analysis with missing modalities. *arXiv preprint arXiv:2505.00568*. 10.48550/arXiv.2505.00568. 1 May 2025. preprint: not peer reviewed.

[14] Van Assen M, Tariq A, Razavi AC, Yang C, Banerjee I, De Cecco CN. Fusion modeling: combining clinical and imaging data to advance cardiac care. Circ Cardiovasc Imaging 2023;16:e014533. 10.1161/CIRCIMAGING.122.014533 PMC10754220 38073535

[15] Jaltotage B, Lu J, Dwivedi G. Use of artificial intelligence including multimodal systems to improve the management of cardiovascular disease. Can J Cardiol 2024;40:1804–1812. 10.1016/j.cjca.2024.07.014 39038650

[16] Farhadizadeh M, Weymann M, Blaß M, Kraus J, Gundler C, Walter S, et al A systematic review of challenges and proposed solutions in modeling multimodal data. *arXiv preprint arXiv:2505.06945*. 10.48550/arXiv.2505.06945. 11 May 2025. preprint: not peer reviewed.

[17] Chen T, Kornblith S, Norouzi M, Hinton G. eds. A simple framework for contrastive learning of visual representations. In: Proceedings of the 37th International Conference on Machine Learning. Vol. 119. Cambridge, MA, USA: PMLR; 2020:1597–1607.

[18] Radford A, Kim JW, Hallacy C, Ramesh A, Goh G, Agarwal S, et al eds. Learning transferable visual models from natural language supervision. In: International Conference on Machine Learning: PmLR; 2021.

[19] You K, Gu J, Ham J, Park B, Kim J, Hong EK, et al eds. CXR-CLIP: toward large scale chest X-ray language-image pre-training. In: International Conference on Medical Image Computing and Computer-Assisted Intervention– MICCAI 2023. Cham, Switzerland: Springer; 2023:101–111.

[20] Tiu E, Talius E, Patel P, Langlotz CP, Ng AY, Rajpurkar P. Expert-level detection of pathologies from unannotated chest X-ray images via self-supervised learning. Nat Biomed Eng 2022;6:1399–1406. 10.1038/s41551-022-00936-9 PMC9792370 36109605

[21] Fu G, Zheng J, Abudayyeh I, Ani C, Rakovski C, Ehwerhemuepha L, et al Cardiogpt: an ecg interpretation generation model. IEEE Access 2024;12:50254–50264.

[22] Yu H, Guo P, Sano A. ECG semantic integrator (ESI): a foundation ECG model pretrained with LLM-enhanced cardiological text. Trans Mach Learn Res 2024;2024:2818. PMC12974696 41816367

[23] Gao Y, Kim S, Austin DE, McIntosh C. MEDBind: unifying language and multimodal medical data embeddings. International Conference on Medical Image Computing and Computer-Assisted Intervention – MICCAI 2024. Vol. 15012. Cham, Switzerland: Springer; 2024:218–228.

[24] Nishihara Y, Nishimori M, Shibata S, Shinohara M, Hirata KI, Tanaka H. Identifying heart failure dynamics using multi-point electrocardiograms and deep learning. Eur Heart J Digit Health 2025;6:447–455. 10.1093/ehjdh/ztaf016 PMC12088712 40395416

[25] Hsiang CW, Lin C, Liu WC, Lin CS, Chang WC, Hsu HH, et al Detection of left ventricular systolic dysfunction using an artificial intelligence-enabled chest X-ray. Can J Cardiol 2022;38:763–773. 10.1016/j.cjca.2021.12.019 35007705

[26] Ueda D, Matsumoto T, Ehara S, Yamamoto A, Walston SL, Ito A, et al Artificial intelligence-based model to classify cardiac functions from chest radiographs: a multi-institutional, retrospective model development and validation study. Lancet Digit Health 2023;5:e525–e533. 10.1016/S2589-7500(23)00107-3 37422342

[27] Liu PY, Lin C, Lin CS, Fang WH, Lee CC, Wang CH, et al Artificial intelligence-enabled electrocardiography detects B-type natriuretic peptide and N-terminal pro-brain natriuretic peptide. Diagnostics (Basel) 2023;13:2723. 10.3390/diagnostics13172723 PMC10487184 37685262

[28] Cho K, Van Merriënboer B, Gulcehre C, Bahdanau D, Bougares F, Schwenk H, et al Learning phrase representations using RNN encoder–decoder for statistical machine translation. In: Moschitti A, Pang B, Daelemans W, eds. Proceedings of the 2014 Conference on Empirical Methods in Natural Language Processing (EMNLP). Doha, Qatar: Association for Computational Linguistics; 2014:1724–1734. .

[29] Chung J, Gulcehre C, Cho K, Bengio Y. Empirical evaluation of gated recurrent neural networks on sequence modeling. *arXiv preprint arXiv:1412.3555*. 10.48550/arXiv.1412.3555. 11 December 2014. preprint: not peer reviewed.

[30] Zhong Z, Zheng L, Kang G, Li S, Yang Y. Random erasing data augmentation. Proc AAAI Conf Artif Intell 2020;34:13001–13008.

[31] Paszke A, Gross S, Massa F, Lerer A, Bradbury J, Chanan G, et al Pytorch: an imperative style, high-performance deep learning library. Adv Neural Inf Process Syst 2019;32:8024–8035.

[32] Dosovitskiy A, Beyer L, Kolesnikov A, Weissenborn D, Zhai X, Unterthiner T, et al. An image is worth 16 × 16 words: transformers for image recognition at scale. In: International Conference on Learning Representations (ICLR); 2021.

[33] Russakovsky O, Deng J, Su H, Krause J, Satheesh S, Ma S, et al Imagenet large scale visual recognition challenge. Int J Comput Vis 2015;115:211–252.

[34] Shi P, Welle MC, Björkman M, Kragic D. Towards understanding the modality gap in clip. In: ICLR 2023 Workshop on Multimodal Representation Learning: Perks and Pitfalls: ICLR; 2023. https://openreview.net/forum?id=8W3KGzw7fNI.

[35] Eslami S, de Melo G. eds. Mitigate the gap: improving cross-modal alignment in CLIP. In: The Thirteenth International Conference on Learning Representations: ICLR; 2025. https://openreview.net/forum?id=aPTGvFqile.

[36] Fahim A, Murphy A, Fyshe A. It's not a modality gap: characterizing and addressing the contrastive gap. *arXiv preprint arXiv:2405.18570*. 10.48550/arXiv.2405.18570. 28 May 2024. preprint: not peer reviewed.

[37] Forrester JS, Diamond G, Chatterjee K, Swan HJ. Medical therapy of acute myocardial infarction by application of hemodynamic subsets (second of two parts). N Engl J Med 1976;295:1404–1413. 10.1056/NEJM197612162952505 790194

[38] Abbas AE, Khoury Abdulla R, Aggrawal A, Crile J, Lester SJ, Boura J. A novel echocardiographic hemodynamic classification of heart failure based on stroke volume index and left atrial pressure. Echocardiography 2017;34:1417–1425. 10.1111/echo.13642 28833458

[39] McDonagh TA, Metra M, Adamo M, Gardner RS, Baumbach A, Bohm M, et al 2021 ESC guidelines for the diagnosis and treatment of acute and chronic heart failure. Eur Heart J 2021;42:3599–3726. 10.1093/eurheartj/ehab853 34922348

[40] Mueller C, McDonald K, de Boer RA, Maisel A, Cleland JGF, Kozhuharov N, et al Heart failure association of the European Society of Cardiology practical guidance on the use of natriuretic peptide concentrations. Eur J Heart Fail 2019;21:715–731. 10.1002/ejhf.1494 31222929

[41] Johnson AEW, Bulgarelli L, Shen L, Gayles A, Shammout A, Horng S, et al MIMIC-IV, a freely accessible electronic health record dataset. Sci Data 2023;10:1. 10.1038/s41597-022-01899-x PMC9810617 36596836

[42] Johnson AEW, Pollard TJ, Berkowitz SJ, Greenbaum NR, Lungren MP, Deng CY, et al MIMIC-CXR, a de-identified publicly available database of chest radiographs with free-text reports. Sci Data 2019;6:317. 10.1038/s41597-019-0322-0 PMC6908718 31831740

[43] Goldberger AL, Amaral LA, Glass L, Hausdorff JM, Ivanov PC, Mark RG, et al PhysioBank, PhysioToolkit, and PhysioNet: components of a new research resource for complex physiologic signals. Circulation 2000;101:E215–E220. 10.1161/01.cir.101.23.e215 10851218

[44] Gow B, Pollard T, Nathanson LA, Johnson A, Moody B, Fernandes C, et al Mimic-iv-ecg: diagnostic electrocardiogram matched subset. Type: Dataset 2023;6:13–14.

[45] Youden WJ . Index for rating diagnostic tests. Cancer 1950;3:32–35. 10.1002/1097-0142(1950)3:1<32::aid-cncr2820030106>3.0.co;2-3 15405679

[46] Gow B, Pollard T, Greenbaum N, Moody B, Johnson A, Herbst E, et al MIMIC-IV-ECHO: Echocardiogram Matched Subset (version 0.1). In: PhysioNet, editor. 2023. 10.13026/ef48-v217.

[47] Matejas J, Żurawski O, Strodthoff N, Lopez Alcaraz JM. Predicting chest radiograph findings from electrocardiograms using interpretable machine learning. *arXiv preprint arXiv:2509.17674*. 10.48550/arXiv.2509.17674, 22 September 2025. preprint: not peer reviewed.

