# Supplementary material for: CardioCLIP: sequential multimodal prediction of echocardiographic abnormalities and natriuretic peptide elevation from electrocardiogram and chest radiographs
Source: Eur Heart J Digit Health. 2026 Oct 1;7(9):ztag158. doi: 10.1093/ehjdh/ztag158 (PMC13695061; doi:10.1093/ehjdh/ztag158)
Supplement: Supplementary file 1 [file ztag158_supplementary_data.pdf]

Supplementary Material for "CardioCLIP: Sequential Multimodal Prediction of Echocardiographic Abnormalities and Natriuretic Peptide Elevation from ECG and Chest Radiographs"

Table of contents	Page
Computational details of CardioCLIP	2-4
Computational details of Sequential Prediction Step	4-6
Clinical Threshold Definitions for Binary Classification	7-9
Analysis of the Time Interval Between ECG/CXR Examinations and Target Data Acquisition	9-13
Threshold-Dependent Performance Metrics at Clinically Relevant LVEF Cutoffs	14-22
References	23-27

Computational details of CardioCLIP

In this study, we propose CardioCLIP, a model based on Contrastive Language-Image Pre-training (CLIP)(1) that takes as input Electrocardiogram (ECG) and chest CXR pairs, encodes them using modality-specific encoders, and learns to minimize the cosine distance between their resulting embeddings. Below, we describe the computational details of the CardioCLIP training methodology.

We denote the i-th ECG data sample as $x_{\mathrm{ECG}}^{i} \in \mathbb{R}^{D\times K}$, where D represents the temporal dimension and K corresponds to the number of ECG leads. In our case, $D = 1000$ and $K = 12$. Similarly, the i-th chest CXR image is represented as $x_{\mathrm{X-ray}}^{i} \in \mathbb{R}^{C\times H\times W}$, where H and W indicate the height and width of the image, respectively, and C denotes the number of channels. In this study, we use $C = 1$, $H = 224$, and $W = 224$.

Each modality is projected into a shared embedding space via modality-specific encoders followed by L2 normalization:

$$z_{\mathrm{ECG}}^{i} = \frac{f_{\mathrm{ECG}}\left(x_{\mathrm{ECG}}^{i}\right)}{\left|f_{\mathrm{ECG}}\left(x_{\mathrm{ECG}}^{i}\right)\right|}, \quad z_{\mathrm{CXR}}^{i} = \frac{f_{\mathrm{CXR}}\left(x_{\mathrm{CXR}}^{i}\right)}{\left|f_{\mathrm{CXR}}\left(x_{\mathrm{CXR}}^{i}\right)\right|}$$

The similarity between ECG and CXR embeddings is defined as:

$$\mathrm{sim}\left(z_{\mathrm{ECG}}^{i}, z_{\mathrm{CXR}}^{i}\right) = {z_{\mathrm{ECG}}^{i}}^{\top} z_{\mathrm{CXR}}^{i}$$

CLIP aligns images and texts in a shared embedding space using a symmetric softmax loss in both directions: image-to-text and text-to-image. However, conventional CLIP tends to form modality-specific clusters in the embedding space(2). To address this modality gap, several methods have been proposed(3, 4). In contrast to datasets containing natural images and diverse captions, ECG and CXR data exhibit limited intra-modality variation. Therefore, separating embeddings of distinct pathological states within the same modality requires stronger constraints.

To this end, we propose a key modification:

Switch Loss: Within each training batch, we randomly select k samples and swap their ECG and CXR embeddings before computing the symmetric softmax loss as in CLIP.

Let the batch size be B (set to 32 in our experiments), and define the set $\mathcal{J} \subset \{1, \dots, B\}$ of k randomly selected indices (with $k = 16$) as:

$$\mathcal{J} \sim Uniform(\mathcal{P}_k(\{1, \dots, B\})), |\mathcal{J}| = k$$

The switch loss for ECG-to-CXR direction is defined as:

$$\mathcal{L}_{\mathrm{ECG}\to\mathrm{CXR}} = \frac{1}{B}\left(\sum_{i\notin\mathcal{J}} \frac{\exp\left(\mathrm{sim}\left(z^{i}_{\mathrm{ECG}}, z^{i}_{\mathrm{CXR}}\right)/\tau\right)}{\sum_{j\notin\mathcal{J}} \exp\left(\mathrm{sim}\left(z^{i}_{\mathrm{ECG}}, z^{j}_{\mathrm{CXR}}\right)/\tau\right) + \sum_{j\in\mathcal{J}} \exp\left(\mathrm{sim}\left(z^{i}_{\mathrm{ECG}}, z^{j}_{\mathrm{ECG}}\right)/\tau\right)}\right.$$

$$\left. + \sum_{i\in\mathcal{J}} \frac{\exp\left(sim\left(z^{i}_{\mathrm{CXR}}, z^{i}_{\mathrm{ECG}}\right)/\tau\right)}{\sum_{j\notin\mathcal{J}} \exp\left(\mathrm{sim}\left(z^{i}_{\mathrm{CXR}}, z^{j}_{\mathrm{CXR}}\right)/\tau\right) + \sum_{j\in\mathcal{J}} \exp\left(\mathrm{sim}\left(z^{i}_{\mathrm{CXR}}, z^{j}_{\mathrm{ECG}}\right)/\tau\right)}\right)$$

Symmetrically, switch loss of the opposite direction is computed as:

$$\mathcal{L}_{\mathrm{CXR}\to\mathrm{ECG}} = \frac{1}{B}\left(\sum_{i\notin\mathcal{J}} \frac{\exp\left(\mathrm{sim}\left(z^{i}_{\mathrm{CXR}}, z^{i}_{\mathrm{ECG}}\right)/\tau\right)}{\sum_{j\notin\mathcal{J}} \exp\left(\mathrm{sim}\left(z^{i}_{\mathrm{CXR}}, z^{j}_{\mathrm{ECG}}\right)/\tau\right) + \sum_{j\in\mathcal{J}} \exp\left(\mathrm{sim}\left(z^{i}_{\mathrm{CXR}}, z^{j}_{\mathrm{CXR}}\right)/\tau\right)}\right.$$

$$\left. + \sum_{i\in\mathcal{J}} \frac{\exp\left(\mathrm{sim}\left(z^{i}_{\mathrm{ECG}}, z^{i}_{\mathrm{CXR}}\right)/\tau\right)}{\sum_{j\notin\mathcal{J}} \exp\left(\mathrm{sim}\left(z^{i}_{\mathrm{ECG}}, z^{j}_{\mathrm{ECG}}\right)/\tau\right) + \sum_{j\in\mathcal{J}} \exp\left(\mathrm{sim}\left(z^{i}_{\mathrm{ECG}}, z^{j}_{\mathrm{CXR}}\right)/\tau\right)}\right)$$

The total loss is given by:

$$\mathcal{L}_{\text{CLIP-Switch}} = \frac{1}{2}\left(\mathcal{L}_{\mathrm{ECG}\to\mathrm{CXR}} + \mathcal{L}_{\mathrm{CXR}\to\mathrm{ECG}}\right)$$

Computational details of Sequential Prediction Step

In the Sequential Prediction Step, a Predictor constructed using a gated recurrent unit (GRU) was trained to sequentially perform binary classification of clinical variables using the pathological embedding vectors obtained in Step 1 as inputs. The Predictor consists of a GRU that updates its hidden state based on the input vectors, followed by a linear layer with a sigmoid activation function that outputs the probability of abnormality.

For a given patient i, let z^{i}_{ECG}, z^{i}_{CXR} denote the embedding vectors obtained from the ECG and CXR, respectively. The initial hidden state of the GRU, denoted as h_{prior}, is

initialized as a zero vector. When ECG is performed first, the hidden state after processing the ECG is computed as

$$h_{ECG} = GRU(z_{ECG}^{i}, h_{prior})$$

The predicted probability of abnormality at this stage is obtained by applying a linear classifier followed by a sigmoid function:

$$p_{ECG} = \sigma(W h_{ECG} + b)$$

Subsequently, when the CXR of the same patient becomes available, the hidden state is updated as

$$h_{ECG\rightarrow CXR} = GRU(z_{CXR}^{i}, h_{ECG})$$

and the final multimodal prediction probability is given by

$$p_{ECG\rightarrow CXR} = \sigma(W h_{ECG\rightarrow CXR} + b)$$

This formulation enables an initial estimation of abnormality using ECG alone, followed by an updated multimodal estimation after incorporating CXR information, thereby supporting ordered computational updating. When the modalities are provided to the model in the reverse order (CXR followed by ECG), the sequence of input embeddings is inverted accordingly.

In the following, we describe the training procedure. Although multiple clinical labels

are used in this study, we present BNP as a representative example.

For patient i, the binary BNP label is defined as

$$y_{BNP}^{i} = \begin{cases} 1 \; if \; BNP > 100 \\ 0 \quad\quad otherwise \end{cases}$$

Other clinical variables are binarized in an analogous manner using clinically meaningful thresholds and are learned within the same framework.

The Predictor is trained using the binary cross-entropy (BCE) loss. Given a predicted probability p and a ground-truth label $y \in \{0,1\}$, the BCE loss is defined as

$$\mathcal{L}_{BCE}(p, y) = -[y \log(p) + (1 - y) \log(1 - p)]$$

For each patient i and each prediction stage $s \in \{ECG, CXR, ECG \to CXR, CXR \to ECG\}$, the loss for BNP prediction is computed as

$$\mathcal{L}_{BNP,s} = \frac{1}{N} \sum_{i=1}^{N} \mathcal{L}_{BCE}(p_{BNP,s}^{i}, y_{BNP}^{i})$$

The total training loss for the Predictor is defined as the sum of the losses across all prediction stages:

$$\mathcal{L}_{Predictor} = \sum_{s} \mathcal{L}_{BNP,s}$$

The same training procedure is applied to other clinical labels by replacing y_{BNP} with the corresponding binary targets. Owing to the recurrent structure of the GRU, this framework can be naturally extended to an arbitrary number of examinations.

Clinical Threshold Definitions for Binary Classification

To perform binary classification for clinical interpretation and model evaluation, we applied established threshold values based on international guidelines and widely cited literature:

Hemoglobin levels <12 g/dL were considered anemic, in accordance with the World Health Organization criteria for adult females and older adults(5).

An HbA1c level >6·5% was used to define diabetes mellitus based on diagnostic criteria from the American Diabetes Association(6).

Blood urea nitrogen (BUN) >20 mg/dL was adopted as a marker of renal dysfunction and has also been used in prognostic scoring systems such as CURB-65(7).

Estimated glomerular filtration rate (eGFR) <60 mL/min/1·73 m^2 indicated moderate-to-severe chronic kidney disease, as defined by the KDIGO guideline(8).

Hyponatremia and hypernatremia were defined as serum sodium <135 mEq/L and >145 mEq/L, respectively, based on clinical norms and thresholds associated with adverse outcomes(9).

Similarly, hypokalemia (K <3·4 mEq/L) and hyperkalemia (K >5·5 mEq/L) thresholds followed values commonly used in studies of electrolyte disorders and emergency management guidelines(10, 11).

A BNP level >100 pg/mL was used to support the diagnosis of heart failure, consistent with the 2021 ESC guidelines(12). A BNP threshold of 400 pg/mL was used to define subsequent severe BNP elevation within 3 years, as values above this level have been reported to be strongly associated with clinically significant heart failure in prior studies(13-15). In the external validation (MIMIC-IV), NT-proBNP >125 pg/mL was defined as abnormal, consistent with the ESC-recommended rule-out threshold for chronic (non-acute) heart failure(16).

Hypertension was defined as systolic blood pressure >140 mmHg or diastolic >90 mmHg, following the JNC-8 criteria(17).

A body mass index (BMI) >25 kg/m^2 was used to indicate overweight, based on the WHO definition(18).

Echocardiographic parameters were dichotomized based on guideline-recommended cutoffs:

- Left ventricular end-diastolic diameter (LVEDD) >55 mm was considered enlarged(19).
- Left ventricular ejection fraction (LVEF) <50% indicated systolic dysfunction(20).
- LV outflow tract velocity-time integral (LVOT VTI) <15 cm was used as a surrogate of low stroke volume, particularly in heart failure with preserved

ejection fraction (21).

- Left atrial volume index (LAVI) >34 mL/m² was used as a marker of left atrial enlargement (19).
- E/e′ ratio >15 was used to suggest elevated left ventricular filling pressure(22).

Valvular heart diseases were classified as significant if the degree of regurgitation or stenosis was moderate or greater, as per ACC/AHA guidelines(23).

A tricuspid regurgitation (TR) pressure gradient >30 mmHg (corresponding approximately to TR velocity >2·8 m/s) was considered indicative of elevated pulmonary pressures(24).

Finally, a stroke volume index (SVI) <35 mL/m² was used to define low-flow states, relevant in both heart failure and valvular heart disease contexts(25).

Analysis of the Time Interval Between ECG/CXR Examinations and Target Data Acquisition

We analyzed the distribution of time intervals between the ECG/CXR examinations and target data acquisition and assessed whether predictive performance varied according to these intervals. The ECG and CXR examinations included in the dataset were performed on the same day. BNP measurements were also obtained on the same day as

the ECG/CXR examinations; therefore, an interval analysis was not performed for BNP. In contrast, echocardiographic parameters, including LVEF and E/e′, were not necessarily obtained on the same day. For model development and evaluation, we used the temporally closest measurement obtained within 14 days before or after the ECG/CXR examination date. The analyses presented in Supplementary Figure 1(A) and Supplementary Table 1 were performed using the entire dataset, combining the training, validation, and test datasets. Supplementary Figure 1(A) shows the distribution of signed calendar-day intervals, defined as the target measurement date minus the ECG/CXR examination date. Negative values indicate that the target measurement preceded the ECG/CXR examination, whereas positive values indicate that it followed the ECG/CXR examination. Supplementary Table 1 summarizes these signed intervals using the mean and the median with the first and third quartiles.

The association between the time interval and predictive performance was evaluated using the area under the receiver operating characteristic curve (AUROC), as shown in Supplementary Figure 1(B). This analysis was performed using only the test dataset. Models trained using measurements obtained within 14 days before or after the ECG/CXR examinations were evaluated by cumulatively restricting test observations according to the maximum absolute calendar-day interval indicated on the x-axis. Thus,

an interval of 0 represents measurements obtained on the same calendar day as the ECG/CXR examination, whereas an interval of *n* represents measurements obtained within *n* days before or after the ECG/CXR examination. The shaded areas represent 95% confidence intervals. No clear material variation in AUROC was observed across the evaluated time intervals.

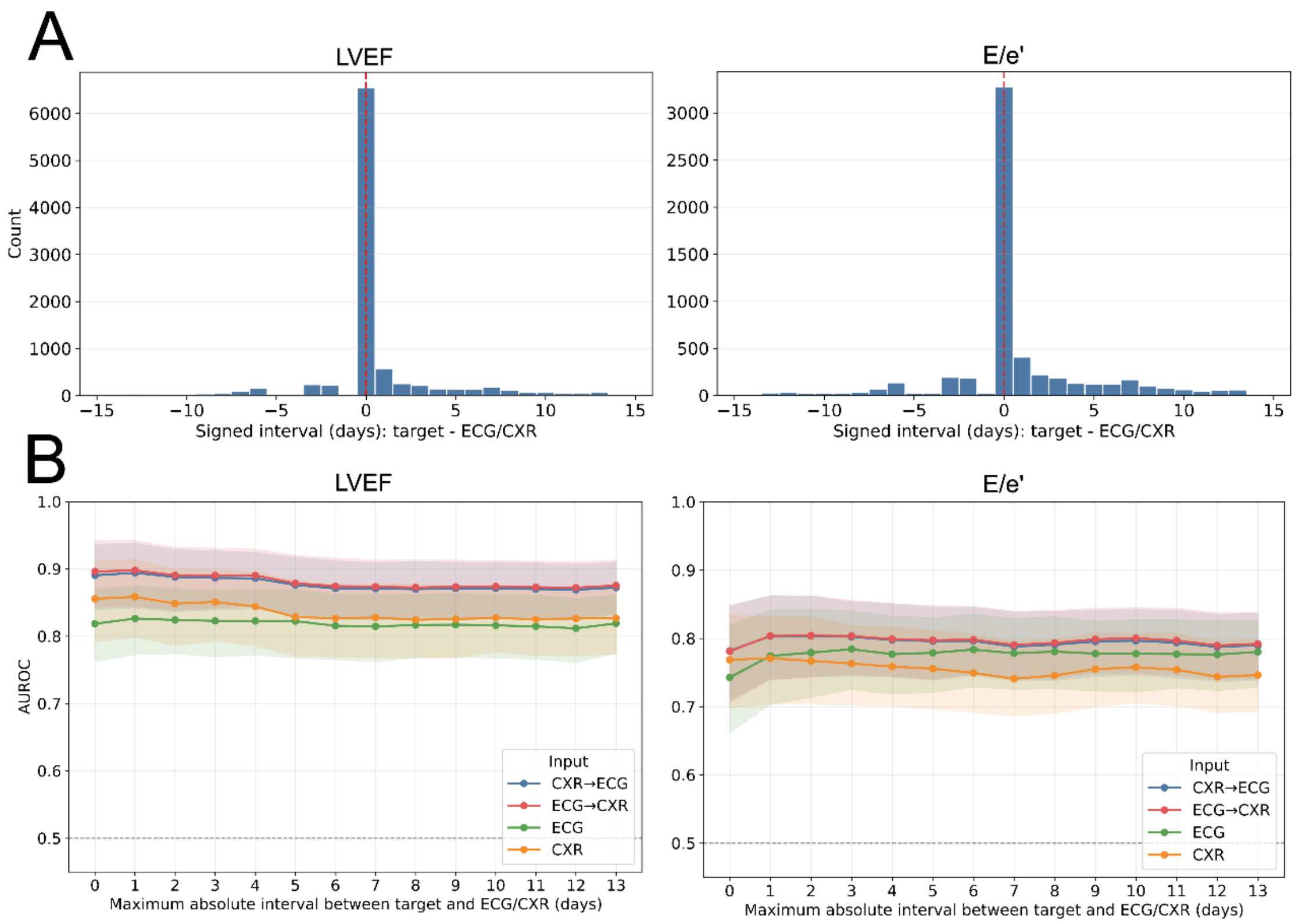

Supplementary Figure 1 | Time intervals between ECG/CXR examinations and echocardiographic data acquisition and their effects on predictive performance. (A) Distributions of the signed time intervals between ECG/CXR examinations and the acquisition of left ventricular ejection fraction (LVEF) and E/e′ data. The signed interval was calculated as the target data acquisition date minus the ECG/CXR examination date; the red dashed line indicates an interval of 0 days. (B) ROC-AUCs for predicting abnormal LVEF and E/e′ using test data acquired within the maximum absolute time interval indicated on the x-axis. Models were trained using data acquired within 14 days before or after the ECG/CXR examination date. Lines represent the input modalities and their sequential order, shaded areas indicate 95% confidence intervals, and the

horizontal dashed line indicates an ROC-AUC of 0.5. CXR, chest radiography; ECG, electrocardiography; LVEF, left ventricular ejection fraction; ROC-AUC, area under the receiver operating characteristic curve.

Supplementary Table 1 | Time intervals between ECG/CXR examinations and acquisition of each target measure.

Time intervals are presented in days as the mean and median with the first and third quartiles [Q1–Q3]. AR, aortic regurgitation; AS, aortic stenosis; BP, blood pressure; E/e′, ratio of early transmitral flow velocity to early diastolic mitral annular velocity; LAVI, left atrial volume index; LVDd, left ventricular end-diastolic diameter; LVEF, left ventricular ejection fraction; LVOT-VTI, left ventricular outflow tract velocity–time integral; MR, mitral regurgitation; BMI, body mass index; SVI, stroke volume index; TRPG, tricuspid; Q1, first quartile; Q3, third quartile;

Measure	Mean	Median [Q1-Q3]
AR severity	0.47	0 [0-0]
AS severity	0.47	0 [0-0]
Diastolic BP	0.73	0 [0-1]
Systolic BP	0.73	0 [0-1]
E/e′	0.73	0 [0-1]
LAVI	0.73	0 [0-1]
LVOT-VTI	0.73	0 [0-1]
LVDd	0.47	0 [0-0]
LVEF	0.46	0 [0-0]
MR severity	0.47	0 [0-0]
BMI	0.67	0 [0-1]
SVI	0.73	0 [0-1]
TRPG	0.52	0 [0-0]

Threshold-Dependent Performance Metrics at Clinically Relevant LVEF Cutoffs

Clinically, an LVEF of <50% was used to identify mildly reduced or reduced LVEF, whereas an LVEF of ≤40% was used to define heart failure with reduced ejection fraction (HFrEF), consistent with the 2021 European Society of Cardiology guidelines(20). Accordingly, separate models were trained using LVEF <50% and LVEF ≤40% as the respective outcomes. For each model, the classification threshold was determined by maximizing the Youden index in the validation set and was subsequently fixed and applied to the independent test set without further optimization. Sensitivity, specificity, positive predictive value, and negative predictive value were calculated in the test set using these fixed thresholds(26). The corresponding 95% confidence intervals were estimated using 2,000 patient-level cluster bootstrap iterations with the percentile method. The results are presented in Supplementary Table 2.

For the prediction of LVEF ≤40%, the CXR→ECG model achieved the highest sensitivity (0.769) and negative predictive value (0.962). For the prediction of LVEF <50%, both the ECG→CXR and CXR→ECG models achieved the highest sensitivity (0.789), with a negative predictive value of 0.930. These results indicate that the sequential multimodal models may have potential as screening tools for identifying patients with reduced LVEF; however, their clinical utility should be interpreted in conjunction with the corresponding

confidence intervals and decision curve analysis findings.

To evaluate the potential clinical utility and incremental value of the multimodal models over the unimodal models, decision curve analysis (DCA) was performed for LVEF ≤40% and LVEF <50% (Supplementary Figure 2). The ECG, CXR, ECG→CXR, and CXR→ECG models were plotted together within the same panel for each LVEF cutoff, allowing direct comparison of their net benefits. The models generally provided a positive net benefit across low-to-moderate threshold probabilities and, over parts of this range, provided greater net benefit than strategies of performing echocardiography in all patients (echocardiography-for-all) or in no patients (echocardiography-for-none). However, the relative net benefit of the multimodal and unimodal models varied according to the threshold probability, and the multimodal models did not demonstrate a consistently greater net benefit across the entire range examined. These findings suggest potential clinical utility as adjunctive screening tools but do not establish uniform incremental clinical value of multimodal prediction over unimodal prediction.

Calibration plots based on the uncalibrated predicted probabilities for LVEF <50% are shown in Supplementary Figure 3. No post hoc calibration was applied. Calibration was assessed using the calibration intercept, calibration slope, and Brier score in addition to visual inspection. The calibration intercepts were −0.059 for ECG, 0.098 for CXR, 0.017

for ECG→CXR, and −0.010 for CXR→ECG. The corresponding calibration slopes were 0.903, 0.728, 0.744, and 0.754, respectively, and the Brier scores were 0.118, 0.139, 0.121, and 0.121, respectively. The calibration intercepts were close to zero, the Brier scores were relatively low, and the predicted probabilities were generally consistent with the observed event rates in the calibration plots. These visual and quantitative assessments indicated generally good calibration across the models. However, the calibration slopes were modestly below 1, indicating a slight deviation from perfect calibration, particularly for the CXR and sequential multimodal models.

Supplementary Table 2 | Classification performance for predicting reduced left ventricular ejection fraction at clinically relevant cutoffs.

Sensitivity, specificity, positive predictive value, negative predictive value, and confusion matrix results are shown for the ECG, CXR, ECG→CXR, and CXR→ECG models using LVEF ≤40% and LVEF <50% as the respective outcomes. Model-specific classification thresholds were determined by maximizing the Youden index in the validation set and were subsequently fixed and applied to the test set without further optimization. The analysis included only test-set samples with valid LVEF measurements; therefore, the sum of TN, FP, FN, and TP is smaller than the total number of patients in the test set. Values in brackets indicate 95% confidence intervals estimated using patient-level cluster bootstrap resampling. Arrows indicate the sequential order of modality input. CI, confidence interval; CXR, chest radiography; ECG, electrocardiography; FN, false negative; FP, false positive; LVEF, left ventricular ejection fraction; TN, true negative; TP, true positive.

**LVEF threshold**	**Model**	**Sensitivity [95% CI]**	**Specificity [95% CI]**	**Positive predictive value [95% CI]**	**Negative predictive value [95% CI]**	**Confusion matrix**			
						**TN**	**FP**	**FN**	**TP**
LVEF ≤ 40%	ECG	0.754 [0.644–0.857]	0.661 [0.623–0.699]	0.203 [0.155–0.256]	0.959 [0.938–0.978]	375	192	16	49

	CXR	0.508 [0.387–0.640]	0.832 [0.801–0.864]	0.258 [0.182–0.336]	0.937 [0.915–0.958]	472	95	32	33
	ECG → CXR	0.554 [0.431–0.681]	0.822 [0.791–0.854]	0.263 [0.192–0.339]	0.941 [0.921–0.962]	466	101	29	36
	CXR → ECG	0.769 [0.667–0.873]	0.674 [0.633–0.712]	0.213 [0.163–0.267]	0.962 [0.942–0.980]	382	185	15	50
LVEF < 50%	ECG	0.732 [0.653–0.811]	0.733 [0.695–0.771]	0.398 [0.333–0.463]	0.919 [0.892–0.945]	373	136	33	90
	CXR	0.715 [0.632–0.791]	0.615 [0.573–0.657]	0.310 [0.257–0.366]	0.899 [0.866–0.929]	313	196	35	88
	ECG → CXR	0.789 [0.714–0.860]	0.676 [0.635–0.715]	0.370 [0.312–0.431]	0.930 [0.903–0.955]	344	165	26	97
	CXR → ECG	0.789 [0.711–0.860]	0.682 [0.641–0.724]	0.375 [0.316–0.437]	0.930 [0.903–0.956]	347	162	26	97

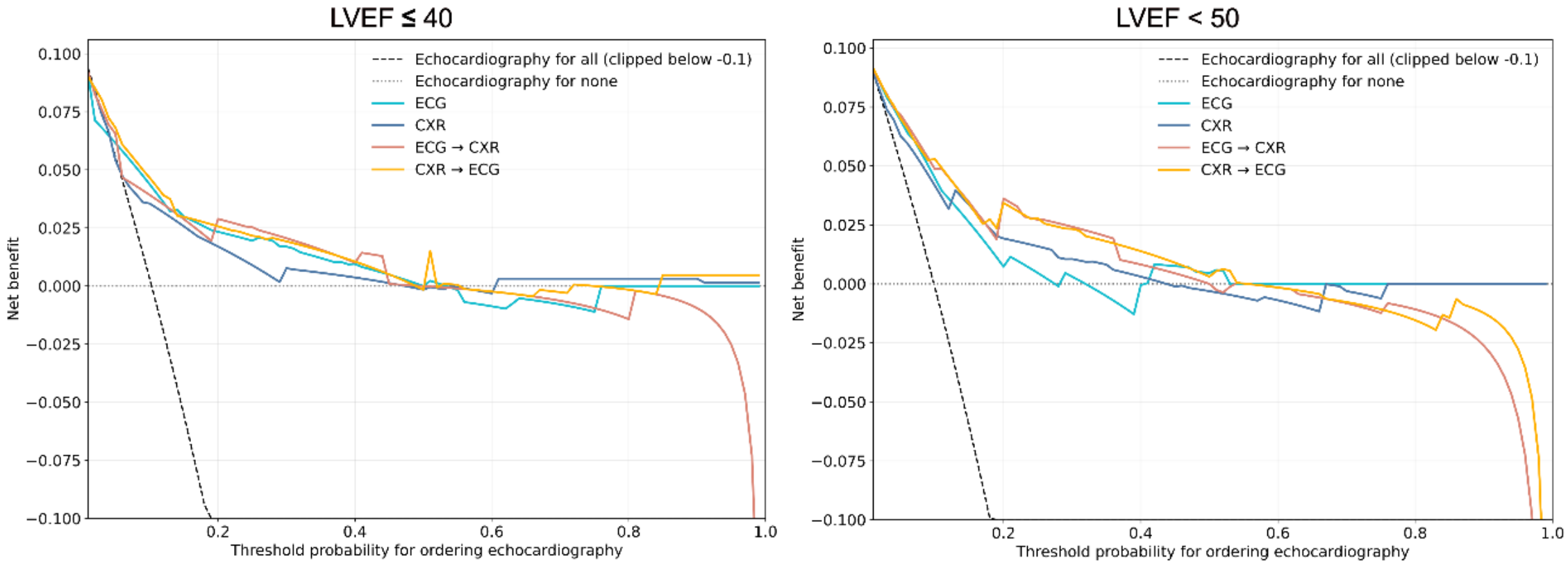

Supplementary Figure 2 | Decision curve analysis for predicting reduced left ventricular ejection fraction.

Decision curve analysis was performed to assess the clinical utility of the ECG, CXR, ECG→CXR, and CXR→ECG models for predicting LVEF ≤40% (left) and LVEF <50% (right). The x-axis represents the threshold probability for ordering echocardiography, and the y-axis represents the net benefit. Colored solid lines indicate the net benefit of each model. The black dashed line represents the strategy of performing echocardiography in all patients (echocardiography-for-all; net-benefit values below −0.1 were clipped), whereas the gray dotted line represents the strategy of performing echocardiography in no patients (echocardiography-for-none). CXR, chest radiography; ECG, electrocardiography; LVEF, left ventricular ejection fraction.

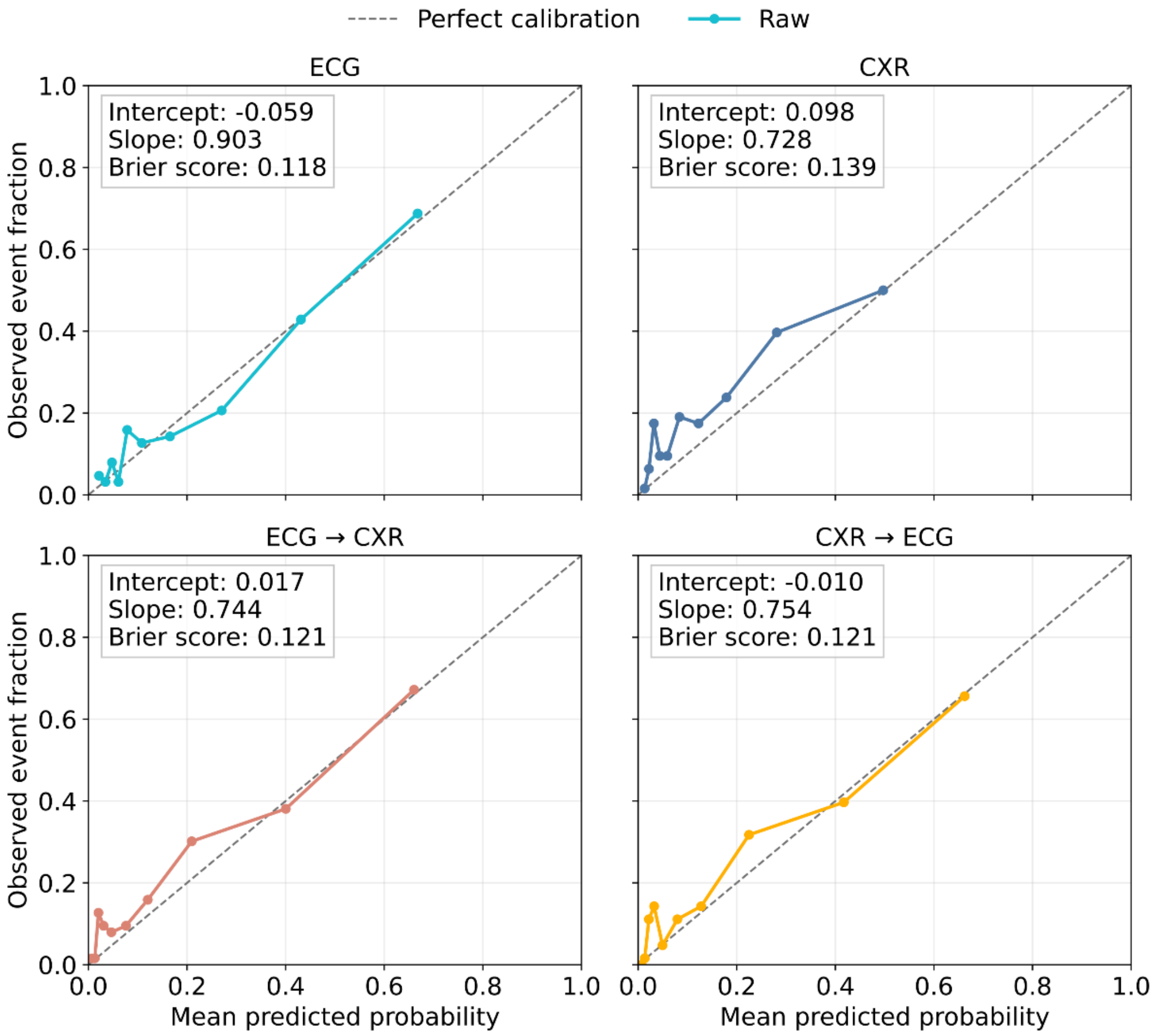

Supplementary Figure 3 | Calibration plots for the prediction of LVEF <50%.

Calibration plots are shown for the ECG, CXR, ECG→CXR, and CXR→ECG models. The x-axis represents the mean predicted probability within each probability bin, and the y-axis represents the corresponding observed event fraction. Colored points and solid lines represent the raw model outputs without post hoc calibration. The gray dashed diagonal line represents perfect calibration, where the predicted probability equals the observed event fraction. The calibration intercept, calibration slope, and Brier score are

reported in each panel. Perfect calibration corresponds to a calibration intercept of 0 and a calibration slope of 1, while a lower Brier score indicates greater accuracy of the predicted probabilities. CXR, chest radiography; ECG, electrocardiography; LVEF, left ventricular ejection fraction.

References

1. Radford A, Kim JW, Hallacy C, Ramesh A, Goh G, Agarwal S, et al., editors. Learning transferable visual models from natural language supervision. International conference on machine learning; 2021: PmLR.

2. Shi P, Welle MC, Björkman M, Kragic D, editors. Towards understanding the modality gap in clip. ICLR 2023 workshop on multimodal representation learning: perks and pitfalls; 2023.

3. Eslami S, de Melo G, editors. Mitigate the gap: Improving cross-modal alignment in CLIP. The Thirteenth International Conference on Learning Representations; 2025.

4. Fahim A, Murphy A, Fyshe A. It's Not a Modality Gap: Characterizing and Addressing the Contrastive Gap. arXiv preprint arXiv:240518570. 2024.

5. Pasricha SR, Rogers L, Branca F, Garcia-Casal MN. Measuring haemoglobin concentration to define anaemia: WHO guidelines. Lancet. 2024;403(10440):1963-6.

6. American Diabetes Association Professional Practice C. 2. Diagnosis and Classification of Diabetes: Standards of Care in Diabetes-2024. Diabetes Care. 2024;47(Suppl 1):S20-S42.

7. Lim WS, van der Eerden MM, Laing R, Boersma WG, Karalus N, Town GI, et

al. Defining community acquired pneumonia severity on presentation to hospital: an international derivation and validation study. Thorax. 2003;58(5):377-82.

8. Levey AS, Eckardt KU, Tsukamoto Y, Levin A, Coresh J, Rossert J, et al. Definition and classification of chronic kidney disease: a position statement from Kidney Disease: Improving Global Outcomes (KDIGO). Kidney Int. 2005;67(6):2089-100.

9. Adrogue HJ, Madias NE. Hyponatremia. N Engl J Med. 2000;342(21):1581-9.

10. Gennari FJ. Hypokalemia. N Engl J Med. 1998;339(7):451-8.

11. Lindner G, Burdmann EA, Clase CM, Hemmelgarn BR, Herzog CA, Malyszko J, et al. Acute hyperkalemia in the emergency department: a summary from a Kidney Disease: Improving Global Outcomes conference. Eur J Emerg Med. 2020;27(5):329-37.

12. McDonagh TA, Metra M, Adamo M, Gardner RS, Baumbach A, Bohm M, et al. 2021 ESC Guidelines for the diagnosis and treatment of acute and chronic heart failure. Eur Heart J. 2021;42(36):3599-726.

13. Prahash A, Lynch T. B-type natriuretic peptide: a diagnostic, prognostic, and therapeutic tool in heart failure. Am J Crit Care. 2004;13(1):46-53; quiz 4-5.

14. York MK, Gupta DK, Reynolds CF, Farber-Eger E, Wells QS, Bachmann KN, et al. B-Type Natriuretic Peptide Levels and Mortality in Patients With and Without Heart Failure. J Am Coll Cardiol. 2018;71(19):2079-88.

15. Taylor CJ, Ordonez-Mena JM, Lay-Flurrie SL, Goyder CR, Taylor KS, Jones NR, et al. Natriuretic peptide testing and heart failure diagnosis in primary care: diagnostic accuracy study. Br J Gen Pract. 2023;73(726):e1-e8.

16. Welsh P, Campbell RT, Mooney L, Kimenai DM, Hayward C, Campbell A, et al. Reference Ranges for NT-proBNP (N-Terminal Pro-B-Type Natriuretic Peptide) and Risk Factors for Higher NT-proBNP Concentrations in a Large General Population Cohort. Circ Heart Fail. 2022;15(10):e009427.

17. James PA, Oparil S, Carter BL, Cushman WC, Dennison-Himmelfarb C, Handler J, et al. 2014 evidence-based guideline for the management of high blood pressure in adults: report from the panel members appointed to the Eighth Joint National Committee (JNC 8). JAMA. 2014;311(5):507-20.

18. Obesity: preventing and managing the global epidemic. Report of a WHO consultation. World Health Organ Tech Rep Ser. 2000;894:i-xii, 1-253.

19. Lang RM, Badano LP, Mor-Avi V, Afilalo J, Armstrong A, Ernande L, et al. Recommendations for cardiac chamber quantification by echocardiography in adults: an update from the American Society of Echocardiography and the European Association of Cardiovascular Imaging. J Am Soc Echocardiogr. 2015;28(1):1-39 e14.

20. Bozkurt B, Coats AJS, Tsutsui H, Abdelhamid CM, Adamopoulos S, Albert N,

et al. Universal definition and classification of heart failure: a report of the Heart Failure Society of America, Heart Failure Association of the European Society of Cardiology, Japanese Heart Failure Society and Writing Committee of the Universal Definition of Heart Failure: Endorsed by the Canadian Heart Failure Society, Heart Failure Association of India, Cardiac Society of Australia and New Zealand, and Chinese Heart Failure Association. Eur J Heart Fail. 2021;23(3):352-80.

21. Omote K, Nagai T, Iwano H, Tsujinaga S, Kamiya K, Aikawa T, et al. Left ventricular outflow tract velocity time integral in hospitalized heart failure with preserved ejection fraction. ESC Heart Fail. 2020;7(1):167-75.

22. Nagueh SF, Smiseth OA, Appleton CP, Byrd BF, 3rd, Dokainish H, Edvardsen T, et al. Recommendations for the Evaluation of Left Ventricular Diastolic Function by Echocardiography: An Update from the American Society of Echocardiography and the European Association of Cardiovascular Imaging. J Am Soc Echocardiogr. 2016;29(4):277-314.

23. Otto CM, Nishimura RA, Bonow RO, Carabello BA, Erwin JP, 3rd, Gentile F, et al. 2020 ACC/AHA Guideline for the Management of Patients With Valvular Heart Disease: Executive Summary: A Report of the American College of Cardiology/American Heart Association Joint Committee on Clinical Practice Guidelines. Circulation.

2021;143(5):e35-e71.

24. Humbert M, Kovacs G, Hoeper MM, Badagliacca R, Berger RMF, Brida M, et al. 2022 ESC/ERS Guidelines for the diagnosis and treatment of pulmonary hypertension. Eur Heart J. 2022;43(38):3618-731.

25. Synetos A, Vlasopoulou K, Drakopoulou M, Apostolos A, Ktenopoulos N, Katsaros O, et al. Impact of Stress Echocardiography on Aortic Valve Stenosis Management. J Clin Med. 2024;13(12).

26. Youden WJ. Index for rating diagnostic tests. Cancer. 1950;3(1):32-5.
